# Deep learning-based phenotype imputation on population-scale biobank data increases genetic discoveries

**DOI:** 10.1038/s41588-023-01558-w

**Published:** 2023-11-20

**Authors:** Ulzee An, Ali Pazokitoroudi, Marcus Alvarez, Lianyun Huang, Silviu Bacanu, Andrew J. Schork, Kenneth Kendler, Päivi Pajukanta, Jonathan Flint, Noah Zaitlen, Na Cai, Andy Dahl, Sriram Sankararaman

**Affiliations:** 1 Computer Science Department, UCLA, Los Angeles, CA USA; 2 Department of Human Genetics, David Geffen School of Medicine at UCLA, Los Angeles, CA USA; 3 Helmholtz Pioneer Campus, Helmholtz Zentrum München, Neuherberg, Germany; 4 Computational Health Centre, Helmholtz Zentrum München, Neuherberg, Germany; 5 School of Medicine, Technical University of Munich, Munich, Germany; 6 Virginia Institute for Psychiatric and Behavioral Genetics and Department of Psychiatry, Virginia Commonwealth University, Richmond, VA USA; 7 Institute of Biological Psychiatry, Mental Health Center - Sct Hans, Copenhagen University Hospital, Copenhagen, Denmark; 8 Neurogenomics Division, The Translational Genomics Research Institute (TGEN), Phoenix, AZ USA; 9 Section for Geogenetics, GLOBE Institute, Faculty of Health and Medical Sciences, Copenhagen University, Copenhagen, Denmark; 10 Institute for Precision Health, David Geffen School of Medicine at UCLA, Los Angeles, CA USA; 11 Neurology Department, UCLA, Los Angeles, CA USA; 12 Section of Genetic Medicine, University of Chicago, Chicago, IL USA; 13 Department of Computational Medicine, UCLA, Los Angeles, CA USA

**Keywords:** Data mining, Genome-wide association studies, Software

## Abstract

Biobanks that collect deep phenotypic and genomic data across many individuals have emerged as a key resource in human genetics. However, phenotypes in biobanks are often missing across many individuals, limiting their utility. We propose AutoComplete, a deep learning-based imputation method to impute or ‘fill-in’ missing phenotypes in population-scale biobank datasets. When applied to collections of phenotypes measured across ~300,000 individuals from the UK Biobank, AutoComplete substantially improved imputation accuracy over existing methods. On three traits with notable amounts of missingness, we show that AutoComplete yields imputed phenotypes that are genetically similar to the originally observed phenotypes while increasing the effective sample size by about twofold on average. Further, genome-wide association analyses on the resulting imputed phenotypes led to a substantial increase in the number of associated loci. Our results demonstrate the utility of deep learning-based phenotype imputation to increase power for genetic discoveries in existing biobank datasets.

## Abstract (web summary)

AutoComplete is a deep learning-based method that imputes missing phenotypes in population-scale biobank datasets, increasing effective sample sizes and improving power for genetic discoveries in genome-wide association studies.

## Main

The past decade has seen the growth of datasets that collect deep phenotypic and genomic data across large numbers of individuals. Although these population-scale biobanks aim to capture a wide range of phenotypes across the population (including demographic information, laboratory tests, imaging, medication usage and diagnostic codes), phenotypes in this setting are frequently missing across many of the individuals for reasons such as cost or difficulty of acquisition (for example, phenotypes derived from imaging scans and other potentially invasive procedures). As a result, our ability to study clinically relevant phenotypes or diseases using biobank data remains limited.

The ubiquity of missing data in the biomedical domain has motivated extensive work into statistical methods for imputing or ‘filling-in’ missing data^1–7^ (see Supplementary Note Section S1 for additional related work). Accurate imputation of large numbers of phenotypes and individuals in population-scale biobank data presents several challenges. First, accurate imputation requires faithfully modeling the dependencies across the phenotypes. Such dependencies can arise because of genetic or environmental effects that are shared across phenotypes. Accumulating evidence for the abundance of shared genetic effects (pleiotropy) even among seemingly unrelated phenotypes suggests that the ability to model dependencies across large numbers of collected phenotypes could substantially improve imputation accuracy. Second, patterns of missingness in these datasets tend to be complex (for example, individuals who were not administered a questionnaire will be missing for all answers relevant to the questionnaire). Third, the method needs to be scalable. Thus, methods that can accurately impute phenotypes in the presence of complex patterns of missingness while being scalable are needed.

Here we propose AutoComplete, a deep-learning method based on an autoencoder architecture designed for highly incomplete biobank-scale phenotype data. Our use of deep learning for imputation is motivated by the ability of neural networks to learn potentially complex dependencies among phenotypes, as shown in the application of neural networks to other biological datasets^8–12^. Earlier works, however, have relied on access to individuals with no missing phenotypes to learn the imputation model^13^ (such an approach would substantially reduce the data available to learn the model) or have assumed that entries in a dataset are missing completely at random^14,15^. To be able to impute in the presence of realistic patterns of missingness, we employed copy-masking, a procedure that propagates missingness patterns present in the data^6^. AutoComplete can impute both binary and continuous phenotypes while scaling with ease to datasets with half a million individuals and millions of entries.

We compared the accuracy of AutoComplete with state-of-the-art missing data imputation methods on two collections of phenotypes derived from the UK Biobank (UKBB)^16^: a set of 230 cardiometabolic-related phenotypes and a set of 372 phenotypes related to psychiatric disorders, each measured across ~300,000 unrelated white British individuals. AutoComplete improved squared Pearson correlation (${r}^{2}$) by 18% on average over the next best method (SoftImpute^5^) and 45% on average for binary phenotypes. AutoComplete is suitable for large-scale biobanks, demonstrating an empirical run time of one hour to fit and impute either dataset. We explored the utility of our method in increasing the power to detect genetic associations for three phenotypes—direct bilirubin, LifetimeMDD^17^ and cannabis ever taken—that had a substantial proportion of missing entries (21%, 80% and 67%, respectively) and were imputed with adequate accuracy in simulations, and for which genome-wide association results could be further verified with studies of comparable phenotypes that did not overlap UKBB. We demonstrate that genome-wide association studies (GWAS) on the imputed phenotypes yield associations that have consistent effects both in the originally observed phenotypes in UKBB and in the external studies. Beyond the replication of significantly associated variants, the polygenic architecture of the imputed phenotypes is highly concordant with those of the originally observed phenotypes in UKBB and the phenotypes measured in the external studies (quantified by their genetic correlation). We observed an increase in effective sample size of 1.8-fold on average, with GWAS on the resulting imputed phenotypes leading to the discovery of 57 new loci. Our results illustrate the value of deep learning-based imputation for genomic discovery.

## Results

### Methods overview

**Fig. 1:**
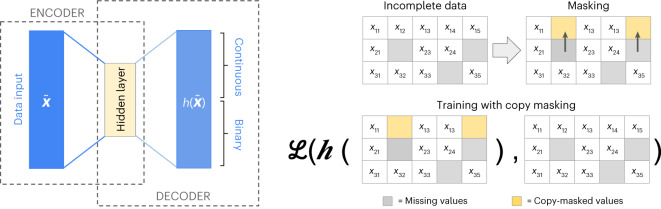
The architecture of AutoComplete. AutoComplete defines a feed-forward encoder-decoder architecture *h* trained using copy-masking, a procedure that simulates realistic missingness patterns that the model uses to impute missing values. AutoComplete minimizes the loss function ${\mathscr{L}}$ that is defined over the observed and masked values. AutoComplete supports the imputation of continuous and binary features.

AutoComplete is based on an autoencoder (a type of neural network) that is capable of simultaneously imputing continuous and binary-valued features. Given a vector of features that represent the phenotypes measured on an individual (some of which might be missing), AutoComplete maps the features to a hidden representation using a nonlinear transformation (encoder), which is then mapped back to the original space of features to reconstruct the phenotypes (decoder). In this process, AutoComplete imputes missing phenotypes (Fig. 1).

AutoComplete aims to learn the autoencoder by masking features that are originally observed in the data and searching for the parameters of the autoencoder that can reconstruct the masked and observed features with minimal error. To enable AutoComplete to impute in the presence of realistic missingness patterns, we employed copy-masking, a procedure that propagates missingness patterns already present in the data^6^.

### Experiment overview

We evaluated the accuracy of phenotypes imputed by AutoComplete on two collections of UKBB phenotypes: a set of 230 cardiometabolic phenotypes derived from patient records and imaging data, and a larger set of 372 phenotypes related to psychiatric disorders from an on-going study of major depressive disorder (MDD)^18^. Each collection contains phenotypes measured across ~300,000 unrelated individuals of white British ancestry, where the median missingness rates across phenotypic entries were 47% and 67% (Supplementary Table 1). The phenotypes in each dataset were collected based on general guidance received from experts with an interest in cardiometabolic and psychiatric disorders, respectively. A focus was placed on phenotypes that were highly missing and of clinical relevance such that imputation would provide a clear utility.

We compared the accuracy of AutoComplete with a representative selection of imputation methods that could be applied at scale. We considered K-Nearest Neighbors (KNN), missForest^19^ and MICE^3^, among the most widely used imputation methods routinely available in data science packages^4^. We also considered SoftImpute^5^ based on its consistently high imputation accuracy in previous works^6,20^. Finally, we also evaluated two recent deep learning-based imputation methods: a generative-adversarial imputation method, GAIN^21^, and a deep generative model, HI-VAE^20^ (see Supplementary Note Section S1 for a more detailed description of related methods).

In determining which methods scale and would therefore be suitable for practical use for the datasets of interest, we assessed the capability of each method to impute the psychiatric disorder dataset in a given amount of time (Supplementary Fig. 1 and Supplementary Note Section S3). Of the considered methods, we determined that missForest and MICE would not be suitable for the scale of our datasets and these were excluded from our large-scale analysis. We also evaluated our method on a small-scale dataset consisting of 86 phenotypes and 50,000 individuals sub-sampled from the cardiometabolic dataset, allowing comparisons with KNN, MissForest^19^ and MICE^3^ (Supplementary Note Section S5).

To quantify the accuracy of each method to impute previously unseen individuals, we adopted a 50% train–test split of the two datasets such that all hyperparameter tuning and training were performed on the training set, whereas evaluations of all methods were performed on the test set (Methods).

To evaluate the imputation methods, we simulated missing entries by masking originally observed phenotypes across a range of missingness levels (1–50%). We examined ${r}^{2}$ between imputed and originally observed values as the primary metric, given its compatibility with continuous and binary phenotypes and its interpretation in terms of the effective sample size^22^. We additionally examined imputation accuracy of binary phenotypes using ${r}^{2}$, area under the precision-recall curve (AUPR) and the area under the receiver operating characteristic curve (AUROC). For each metric, we quantified standard error and confidence intervals using 50 bootstrap replicates. To test for significant differences in the imputation accuracy obtained by each method, we performed a two-tailed significance test using the bootstrap standard errors.

We explored the utility of phenotypes imputed using AutoComplete for improving power in GWAS for three phenotypes: direct bilirubin, LifetimeMDD and cannabis ever taken. To account for imputation uncertainty, we implemented a bootstrapping procedure to produce ten multiple imputations and combined our results across these multiple imputations (Methods). To determine whether using AutoComplete for downstream analysis leads to reliable biological discoveries, we examined the consistency of effects at individual loci found to be significantly associated with the imputed phenotype and the similarity of the polygenic architecture of the imputed phenotype. We performed these analyses both within UKBB (comparing the imputed portion of a phenotype with its originally observed portion) and by comparing the UKBB imputed phenotypes with external GWAS that do not overlap with UKBB.

### AutoComplete significantly improves imputation accuracy

**Fig. 2:**
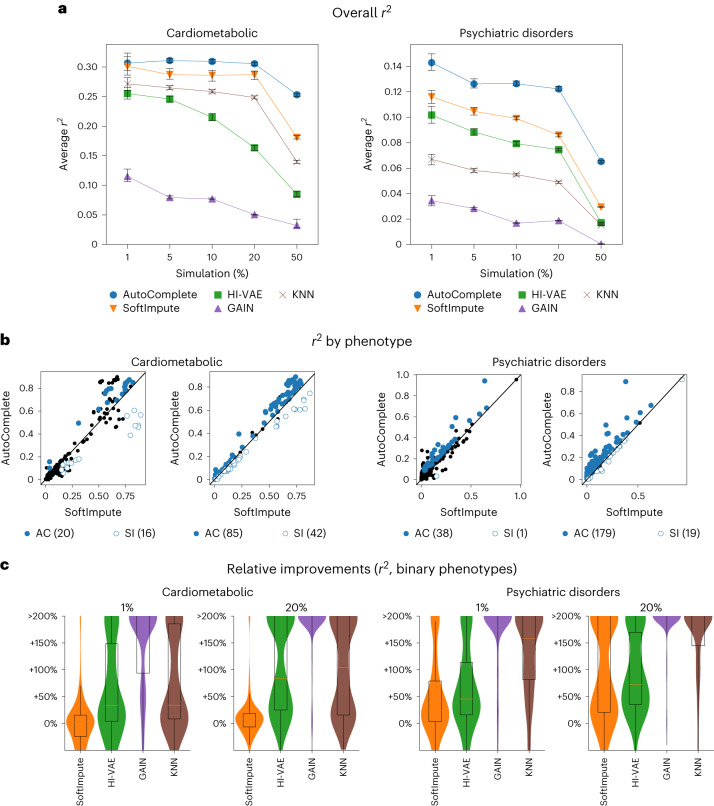
AutoComplete provides accurate imputations across a range of simulation settings. **a**, Average Pearson’s *r*^*2*^ imputation accuracy across phenotypes for a range (1–50%) of simulated missingness (bars denote 95% CIs obtained through 100 bootstraps). **b**, Comparisons of imputation accuracy per phenotype between AutoComplete (AC) and SoftImpute (SI; next best). Blue dots indicate a significant difference in accuracy (two-sided *t*-test with$P < 2.17\times 1{0}^{-4}$ and $P < 1.34\times 1{0}^{-4}$, adjusted for the number of phenotypes, for cardiometabolic and psychiatric disorder phenotypes). **c**, Relative improvements in imputation accuracy for binary-valued phenotypes between AutoComplete and each compared method (percentages thresholded at 200% for clarity). Boxes indicate the first, median and third quartiles, and whiskers extend to 1.5× the interquartile range. The psychiatric disorders dataset contained 372 phenotypes and the cardiometabolic dataset contained 230 phenotypes.

AutoComplete obtained the most accurate imputations across all levels of missingness (from 1% to 50%) in the tested datasets (Table 1 and Fig. 2). Imputation accuracy was generally higher in the cardiometabolic dataset relative to the psychiatric disorders dataset, which we hypothesize can be attributed, in part, to the greater proportion of missing entries in the latter (Supplementary Table 1). Further, the imputation accuracy of all methods decreased with increasing levels of missingness. Although SoftImpute (based on a linear model) was most accurate among the existing methods, AutoComplete obtained the highest overall accuracy with an average improvement over SoftImpute of 18% ($P=1.21\times 1{0}^{-67}$ under two-tailed *t*-test). Separately for the cardiometabolic and psychiatric disorder datasets, AutoComplete obtained improvements of 11% and 25% ($P=3.54\times 1{0}^{-26}$ and $P=2.28\times 1{0}^{-301}$) respectively, indicating the value of modeling nonlinear relationships among phenotypes (Fig. 2).

**Table 1:** Summary of imputation accuracy

	Cardiometabolic				Psychiatric disorders			
	*r*^2^	*r*^2^ binary	AUPR	AUROC	*r*^2^	*r*^2^ binary	AUPR	AUROC
GAIN	0.071 (0.002)	0.015 (0.002)	0.245 (0.004)	0.587 (0.007)	0.020 (0.000)	0.013 (0.001)	0.281 (0.001)	0.428 (0.001)
KNN	0.237 (0.002)	0.025 (0.001)	0.259 (0.004)	0.600 (0.003)	0.049 (0.001)	0.041 (0.001)	0.398 (0.001)	0.596 (0.001)
HI-VAE	0.193 (0.002)	0.067 (0.003)	0.337 (0.001)	0.693 (0.001)	0.072 (0.001)	0.070 (0.001)	0.430 (0.001)	0.696 (0.001)
SoftImpute	0.269 (0.003)	0.064 (0.002)	0.327 (0.006)	0.689 (0.007)	0.087 (0.001)	0.071 (0.001)	0.425 (0.002)	0.658 (0.002)
AutoComplete	0.297 (0.002)	0.096 (0.004)	0.361 (0.006)	0.726 (0.005)	0.112 (0.001)	0.099 (0.001)	0.450 (0.002)	0.701 (0.001)

Average metrics across all simulations (1%, 5%, 10%, 20% and 50% missing data) are shown for Cardiometabolic and Psychiatric disorder phenotypes. We report the correlation coefficient (*r*^2^), the *r*^2^ restricted to binary-valued phenotypes (*r*^2^ binary), and AUPR and AUROC for binary-valued phenotypes. Standard errors are shown in parentheses.

AutoComplete significantly improved ${r}^{2}$ for 20 (85) phenotypes over SoftImpute with 1% (20%) missingness in the cardiometabolic dataset (*P* *<* 0.05/230 correcting for the number of phenotypes tested). Analogously, AutoComplete significantly improved ${r}^{2}$ for 36 (179) phenotypes with 1% (20%) missingness in the psychiatric disorders dataset (Supplementary Table 2; *P* *<* 0.05/372 correcting for the number of phenotypes tested), where the number of phenotypes on which AutoComplete improved accuracy was greater than those where it had lower accuracy in all settings (Supplementary Table 2).

The improvements in imputation accuracy were particularly substantial for binary phenotypes. Here, AutoComplete obtained a relative improvement over the next best method (SoftImpute) of 51% in ${r}^{2}$ on the cardiometabolic data and 39% on the psychiatric disorders data across all simulations (Fig. 2c). We found qualitatively similar trends for other metrics such as AUPR and AUROC (Supplementary Table 2). In comparison with SoftImpute, AutoComplete imputation obtained a relative increase in AUPR of 10% and AUROC of 5% in the cardiometabolic dataset and an increase of 6% and 7% for both metrics in the psychiatric disorders dataset (Table 1).

We performed a separate experiment on a small-scale subset of UKBB in which we compare AutoComplete with missForest and MICE, which could not scale to the full UKBB phenotypes, and found that AutoComplete remains the most accurate method in this setting (Supplementary Fig. 4 and Supplementary Note Section S5).

Finally, we also explored the importance of the copy-masking procedure to the accuracy of AutoComplete. We compared AutoComplete trained with copy-masking and a denoising autoencoder trained with uniformly random masking (Supplementary Note Section S6). For the setting of 1% missingness, the highest average ${r}^{2}$ obtained through uniformly random masking was 0.121 compared with 0.142 with AutoComplete (15% lower with uniformly random masking) with similar trends in tests with increasing missingness (average 16% improvement using copy-masking; Supplementary Fig. 5 and Supplementary Note Section S6). We further assessed the importance of copy-masking in the evaluation step used to measure imputation accuracy. Instead of copying existing missing patterns, we chose values to be missing uniformly at random among all observed values until 1–50% of the observed data was withheld for imputation(Supplementary Fig. 6 and Supplementary Note Section S6). When not propagating the existing missing data patterns for testing, the imputation accuracy (*r*^2^) of AutoComplete was inflated to 0.164 on average (0.117 originally), whereas the imputation accuracy of LifetimeMDD grew to 0.757 (0.407 originally) across 1–50% simulations. We therefore conclude that copy-masking is integral to evaluating imputation accuracy and that AutoComplete benefits from mimicking realistic missingness patterns that aid the denoising behavior of the deep-learning model.

### Imputed phenotypes lead to replicable genomic discoveries

We explored the utility of phenotypes imputed using AutoComplete for improving power in GWAS. We selected three phenotypes (direct bilirubin, LifetimeMDD and cannabis ever taken) that had a considerable fraction of missing entries (21%, 80% and 67%, respectively) and were imputed with reasonable accuracy in simulations (${r}^{2}$ = 0.510, 0.507 and 0.310, respectively). To confirm that these phenotypes are accurately imputed in real data, we verified imputation quality measured as the ratio of the variance between the imputed portion of the phenotype and the variance of the observed portion (analogous to the metrics used to measure the quality of genotype imputation^23,24^) was sufficiently high across the three phenotypes (0.21, 0.52 and 0.28, respectively). The type of each phenotype differed, where direct bilirubin was continuous, cannabis ever taken was ordinal and LifetimeMDD was binary. Both direct bilirubin and cannabis ever taken were estimated as continuous phenotypes by AutoComplete, whereas LifetimeMDD was estimated as a binary phenotype in a continuous probability scale from 0 to 1. For the purpose of concise downstream analysis, all three phenotypes were treated as continuous phenotypes. Importantly, each of these phenotypes had sufficiently large GWAS summary statistics that did not overlap with UKBB. Furthermore, we implemented a bootstrapping procedure to produce ten multiple imputations to account for uncertainties that arise during the imputation process. We then combined genetic analyses across the multiple imputations using Rubin’s rule (Methods).

**Fig. 3:**
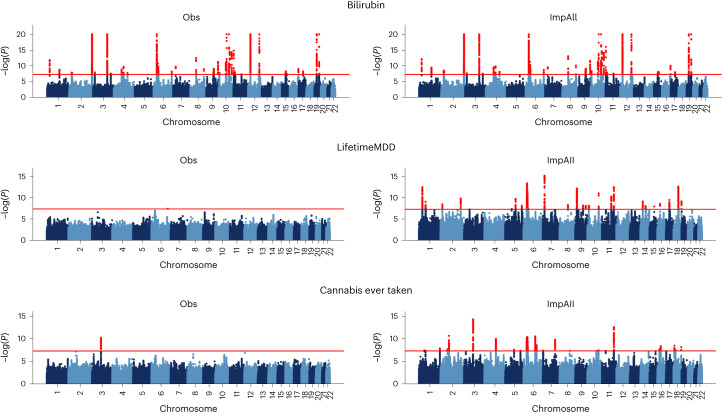
Imputation with AutoComplete increases GWAS power. Results of GWAS of the observed portions of bilirubin, LifetimeMDD and cannabis ever taken in the UKBB (indicated as Obs), where each phenotype had 21%, 80% and 67% missingness respectively. GWAS was then performed for all individuals in the dataset after using AutoComplete to impute the missing entries for each phenotype (indicated as ImpAll). The significance threshold of $P < 5\times 1{0}^{-8}$ is indicated by a red line, and SNPs passing the threshold are highlighted in red.

We estimated the effective gain in sample size resulting from imputation using AutoComplete for each phenotype. We observed an increase in sample size of around 1.8-fold on average: LifetimeMDD had an effective sample size of 193,379 from 67,164 original samples, a 1.87-fold increase, whereas bilirubin had a 0.13-fold increase consistent with the lower missingness rate (Supplementary Table 4 and Methods). We performed GWAS on each of the imputed phenotypes and observed 57 new significantly associated loci in total: 28 each for LifetimeMDD and cannabis ever taken and one new locus for bilirubin, consistent with the missingness rates across these phenotypes (Fig. 3 and Table 2).

**Table 2:** Significantly associated loci from GWAS analysis of three phenotypes of interest and increase in the number of hits through the use of imputed phenotypes

Phenotype	Missing (%)	AutoComplete (Imp) no. of loci	Observed no. of loci	AutoComplete (ImpAll) no. of loci	More no. of loci
Bilirubin	21	17	42	43	1
LifetimeMDD	80	23	1	29	28
Cannabis ever taken	67	11	1	29	28

GWAS was performed on three phenotypes of interest on the observed, imputed (Imp) and the cohort of all individuals including imputed missing observations (ImpAll). The number of additionally discovered loci (More no. of loci) in applying AutoComplete were tallied in comparison with the original phenotype without imputation.

To assess the reliability of phenotypes imputed using AutoComplete for GWAS, we performed GWAS on only the imputed portions of the phenotypes in UKBB (termed Imp). For each of the three phenotypes, we examined the consistency of effects at individual loci found to be significantly associated with the imputed phenotype and the similarity of the polygenic architecture of the imputed phenotype. We performed these analyses by comparing the results obtained from the imputed UKBB phenotypes (Imp) with the observed phenotypes within UKBB (Obs) and to external studies that do not overlap with UKBB (Ext). We analyzed four external cohorts: bilirubin from the Vanderbilt University Medical Center (VUMC)^25^, major depression from the Psychiatric Genomic Consortium (PGC)^26^ and from 23andMe (23andMe)^17^, and lifetime cannabis use from the International Cannabis Consortium (ICC)^27^.

**Fig. 4:**
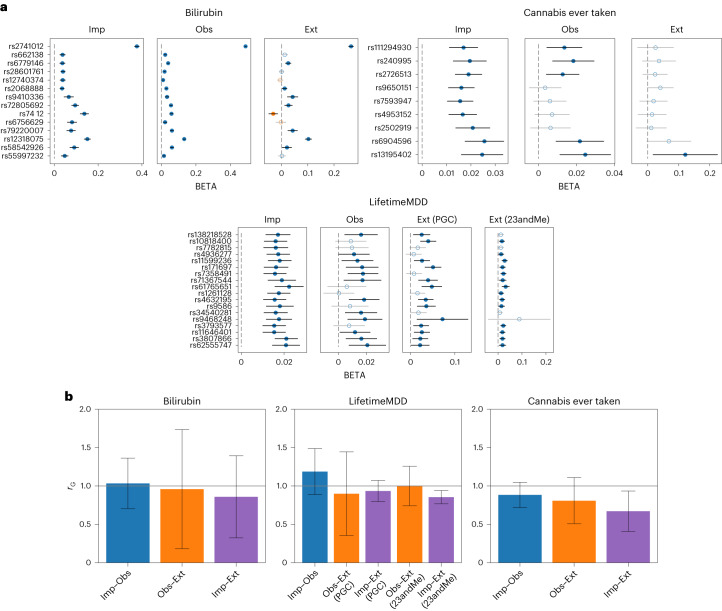
Analysis of the genetic architecture of phenotypes imputed with AutoComplete. **a**, Effect sizes of significantly associated loci based on imputed phenotypes were examined in the association studies of the observed phenotypes in UKBB (Obs or observed) and comparable nonUKBB studies (Ext or external). Genome-wide analysis was performed across 5,776,313 SNPs. For imputed phenotypes, circles indicate the mean effect based on multiple imputation (black bars indicate the 95% CI). Mismatches in effect directions are highlighted in orange. Effects that were not significantly different from zero in Obs or Ext (at *P* < 0.05, two-sided *t*-test) are denoted using empty markers. Loci are visualized that were present across compared studies for each phenotype. **b**, Genetic correlation (${r}_{G}$) for bilirubin, LifetimeMDD and cannabis ever taken between UKBB observed and imputed (Obs and Imp, in blue) phenotypes and nonUKBB cohorts (Ext, in orange and purple). Bar heights for genetic correlations that involve imputed phenotypes indicate mean ${r}_{G}$ based on multiple imputation. Black bars indicate the 95% CI.

We performed GWAS on each of the three Imp phenotypes within UKBB to detect 51 significantly associated loci ($P < 5\times 1{0}^{-8}$). For each of the significant loci, we first examined the concordance of its effect direction in the phenotypes originally observed in UKBB (Obs). Of the 51 loci, we specifically inspected 38 Obs loci that demonstrated effect sizes significantly distinct from zero (*P* < 0.05). All 38 loci had a matching direction of effects in the corresponding Obs phenotype (*P* = 7.3 × 10^−12^ for a binomial test; Fig. 4a and Table 3). We then performed the same validation procedure given summary statistics of the Ext phenotypes. Of the 51 loci, 43 could be located in the summary statistics of the nonUKBB studies, of which 26 loci had effects significantly distinct from zero (*P* < 0.05). Of the 26 loci, 25 had matching direction of effects (96%; *P* = 8.0 × 10^−7^ for a binomial test; Fig. 4a and Table 3). We observed that bilirubin was the only phenotype in which the direction of effects did not match across all associated loci, with 8 of 9 loci having consistent direction of effects. However, this rate is consistent with the rate of sign consistency that we observe for loci discovered to be associated with originally observed bilirubin (14 of 15; Table 3). We further report the number of matching effects regardless of being significantly different from zero in Supplementary Table 5. We observed qualitatively similar results when testing the *P* values of the discovered loci in both the Obs and Ext datasets: 38 of 51 loci had *P* < 0.05 in the Obs dataset, whereas 28 of 43 had *P* < 0.05 in the Ext dataset (compared with 15 of 33 for loci discovered in the Obs dataset; Table 3).

**Table 3:** Comparison of GWAS performed on three phenotypes of interest and replicability of the significantly detected loci

Replication within UKBB (imputed versus observed)		
	Imputed–Observed	
Phenotype	Effect	Sig
Bilirubin	17 of 17	17 of 17
LifetimeMDD	14 of 14	14 of 23
Cannabis ever taken	7 of 7	7 of 11

Replication of UKBB associations (observed and imputed) in external datasets					
		Observed–External		Imputed–External	
Phenotype	Study	Effect	Sig	Effect	Sig
Bilirubin	ref. ^25^	14 of 15	15 of 32	8 of 9	9 of 14
LifetimeMDD	ref. ^26^	0 of 0	0 of 1	13 of 13	13 of 18
LifetimeMDD	ref. ^17^	0 of 0	0 of 1	16 of 16	16 of 20
Cannabis ever taken	ref. ^27^	0 of 0	0 of 0	1 of 1	1 of 9

The significantly detected loci in one study were compared with another in terms of how many loci match in effect direction of the SNP (Effect) where effect size was distinct from zero in the replication study (*P* < 0.05), and how many were marginally significant (Sig) overall (*P* < 0.05). Significance was determined using a two-sided *t*-test. Comparisons were performed between imputed and originally observed phenotypes in UKBB first (upper) and then between the associations detected in the former two and association studies external to UKBB (lower). Denominator indicates the total number of SNPs present in the compared study and matching the criteria for comparison.

We measured the similarity in genome-wide SNP effects between the imputed (Imp), observed (Obs) and external (Ext) phenotypes by estimating the genetic correlation (${r}_{G}$) of their summary statistics using LD score regression (LDSC)^28^. The average ${r}_{G}$ between Imp and Obs phenotypes was 1.03 (95% confidence intervals (CIs) overlap 1 in all cases; Fig. 4b). When comparing the Imp and corresponding Ext phenotypes, ${r}_{G}$ was 0.83 on average. The lower ${r}_{G}$ (Imp, Ext) is not unexpected given the differences between UKBB and the external studies. For example, the cannabis ever taken phenotype in UKBB takes distinct values based on the number of times cannabis was used (never used, used 1–2, 3–10, 11–100 and more than 100 times), whereas the cannabis usage phenotype measured in ICC was a binary phenotype on whether or not an individual reported using cannabis in their lifetime. The ICC GWAS is a meta-analysis of 13 studies that report a wide range in the prevalence of lifetime cannabis use, reflecting differences across these studies. To place these ${r}_{G}$ estimates in context, we compared the ${r}_{G}$ of pairs of Imp and Ext phenotypes with the corresponding pairs of Obs and Ext phenotypes to find that the two sets of estimates are not significantly different from each other (${r}_{G}$ of 0.92 across the pairs of Obs and Ext phenotypes so that a test of the difference in ${r}_{G}$(Obs, Ext) to ${r}_{G}$(Imp, Ext) failed to reject the null hypothesis of no difference in ${r}_{G}$; Fig. 4b). Taken together, we conclude that the genetic architecture of the imputed phenotypes is similar to that of the originally observed phenotypes both at individual GWAS loci and across the genome.

## Discussion

The ubiquity of missing data in population-scale biobanks necessitates effective methods for imputation. Here, we describe AutoComplete, a deep-learning approach to imputation, which we demonstrate to be accurate and efficient for imputing phenotypes in the UK Biobank.

AutoComplete increased the imputation accuracy of highly missing phenotypes related to cardiometabolic and psychiatric disorders in comparison with state-of-the-art linear methods. This implies that understanding nonlinear dependencies among phenotypes in biobank data is important. Patterns of missingness are often structured for biobank-type data as a consequence of the data-gathering procedures. We also observed that realistic simulations of missing data make a substantial contribution to the accuracy of the model learned for imputation (Supplementary Note Section S6). Our use of copy-masking provides a straightforward and general approach for training deep-learning methods in the presence of complex, structured missingness that can be expanded and adapted to new settings.

For the application of our method to new datasets, it would be important to be able to quantitatively determine the quality of imputations for each phenotype. Given that we were able to validate a set of phenotypes chosen based on the variance ratio of the imputed to the observed phenotype (>0.2), accuracy measured on masked phenotypes (${r}^{2}$ > 0.2) and sufficient fraction of missing entries (>10%), we recommend these metrics as a starting point for future analyses. To allow users to explore choices that might be most appropriate for their specific analyses, we provide the ability for a user of our software package to view these metrics for each phenotype similarly to how we have examined them for any imputed dataset.

We discuss limitations of our method and directions for future work. First, the basic autoencoder architecture underlying our method can be extended in many ways. Although we determined through cross-validation that the majority of the imputation accuracy is gained architecturally from the first three layers and the support for continuous and binary imputations, a fuller exploration of the architecture of the neural network could lead to further improvements in accuracy. Second, because biobanks collect diverse data modalities, including imaging, time-series and multiomic data, imputing missing data that arises in the context of these diverse data types remains a challenge. The phenotypes that we impute in our current work are a mix of continuous, binary and ordinal types, wherein we treat ordinal phenotypes as continuous. The modularity of the underlying neural network architecture will enable our method to deal with the diversity of phenotypic data types that are being gathered, and we leave this as a promising direction for future work. Finally, the consequence of using a deep-learning method is that the resulting imputation phenotypes are often challenging to interpret. Such interpretations are critical to understanding whether an imputed phenotype is enriched for the genetic component of the original phenotype. Methodology for interpreting deep-learning methods is an area of active research^29,30^ and could be extended to our setting. Analyzing the signals driving our imputation method when applied to biological datasets could reveal distinct subtypes of a disease and could provide insights into disease etiology. Interpretable components could also give higher credence to the imputed phenotypes.

## Methods

### Datasets

The UKBB^16^ makes available genetic data for up to half a million individuals and thousands of traits. We gathered two collections of phenotypes in UKBB.

We collected a group of 230 cardiometabolic phenotypes^31,32^ consisting of phenotypes and serum biomarkers derived from body imaging and laboratory measurements relevant to cardiometabolic disorders, consumption of prescribed drugs (for example, medication for cholesterol or aspirin), measures of daily physical activity and food consumption, as well as anthropometric and general demographic information. In addition, we collected International Statistical Classification of Diseases and Related Health Problems tenth revision (ICD-10) and ICD-9 codes relating to nonalcoholic fatty liver disease^33,34^, and ICD-10, ICD-9 and Office of Population Censuses and Surveys Classification of Interventions and Procedures version 4 codes relating to coronary artery disease as described^35^.

We constructed a second dataset of 372 phenotypes related to psychiatric disorders. This included lifetime and current MDD symptom screens^36,37^, psychosocial factors, comorbidities, family history of common diseases, a broad range of demographic information, as well as both deep and shallow definitions of MDD derived from symptom questionnaires using clinical diagnostic criteria or self-reports^17^. Both datasets consist of ~300,000 white British unrelated individuals. Each of these collections included a mix of continuous and binary-valued phenotypes (Supplementary Table 1). Missingness rates for phenotypes across individuals varied from 0% (age, sex) and up to 99% (addiction, self-harm).

For each dataset containing *N* individuals and *P* phenotypes, a data matrix of dimension *N* × *P* was created including missing values. Approximately 50% of all individuals were reserved for testing (evaluating the accuracy of the methods) and the remainder was used for training and any hyperparameter tuning for all methods (in an 80–20 split). Continuous phenotypes were normalized to have zero mean with unit variance per phenotype. Binary-valued phenotypes were processed specific to the capabilities of each method; for methods that did not handle binary data, labels were converted from 0,1 to −0.5,0.5 and treated as continuous values. To prevent information leakage, statistics of the training split were used to normalize the test split.

### AutoComplete

AutoComplete is based on a type of neural network that is capable of simultaneously imputing continuous and binary-valued phenotypes. For each individual, AutoComplete considers a fixed list of phenotypes including missing values and reconstructs all phenotypes from a latent representation using an autoencoder architecture. Of the input phenotypes, missing entries were masked (set to zero), then all observed phenotype values were transformed to a hidden representation in the encoding stage. The decoding stage transforms the hidden representation back to the input space such that all phenotypes were reconstructed. To support heterogeneous data types, imputed entries corresponding to binary phenotypes were obtained as the output of a sigmoid function so that these entries lie in the range [0,1].

Let $\widetilde{X}$ denote a *N* × *P* phenotype matrix such that ${\widetilde{X}}_{{ij}}$ is the value of the *j*th phenotype measured on the *i*th individual, $M$ denotes a $N\times P$ indicator matrix (termed the Mask matrix) where ${M}_{{ij}}=1$ if the *j*th phenotype is observed for the *i*th individual and ${M}_{{ij}}=0$ otherwise. For simplicity, continuous and binary phenotypes were organized in $\widetilde{X}$ such that the first $C$ phenotypes were continuous.

$h$ denotes the nonlinear function corresponding to the autoencoder. The function $h$ imputes both missing phenotype values and reconstructs observed ones. During imputation, only the imputed missing values are used. Using the LeakyReLU function $\varPhi$ as a nonlinearity in the hidden layer and the sigmoid function $s$ that was applied to binary-valued imputations, we define for the case of one hidden layer the following feed-forward function $h$ (additional hidden layers could be defined analogously):

$${\textbf{h}}^{\left(1\right)}=\varPhi \left({{W}^{\,(1)}\widetilde{{\textbf{X}}}}_{i,:}+{{\textbf{b}}}^{(1)}\right)$$

$${\textbf{h}}^{\left(2\right)}={W}^{\,(2)}{\textbf{h}}^{\left(1\right)}+{{\textbf{b}}}^{(2)}$$

$$\,h\left({\widetilde{{\textbf{X}}}}_{i,:}\right)=\left(\left\{{h}_{j}^{\left(2\right)}\right\}_{j=1,\ldots ,C},\left\{s\left({h}_{j}^{\left(2\right)}\right)\right\}_{j=C+1,\ldots ,P}\right)\,$$

where

$$\varPhi \left(x\right)=\max \left(0,x\right)-{l}_{\varPhi }\min \left(0,x\right),{\rm{and}}$$

$$s\left(x\right)=\frac{1}{1+{\mathrm{e}}^{-x}}\,$$

${\widetilde{{\textbf{X}}}}_{i,:}$ denotes row $i$ of ${\widetilde{{{X}}}}$ (equivalently the vector of phenotypes associated with individual *i*). For each layer, the learnable weight parameter $W$ is a $D\times P$ matrix where *D* is the dimension of the hidden representation, whereas the bias vector ${\textbf{b}}$ is of length *D*.

Given function *h*, the final imputed matrix $\hat{X}$ is constructed from $\widetilde{{{X}}}\,$ as follows:

$${\hat{{\textbf{X}}}}_{i,:}={M}_{i,:}\cdot {\widetilde{{\textbf{X}}}}_{i,:}+(1-M_{i,:})\cdot h\left({\widetilde{{\textbf{X}}}}_{i,:}\right),1 < i < N$$

Here · denotes entrywise product.

In training, we promoted imputation using $h$ such that both truly observed and masked phenotype values were subject equally to a reconstruction loss. Observed values were withheld based on existing missingness patterns, which were randomly drawn from the dataset and then applied to other individuals—a process we refer to as copy-masking. To do this, a binary mask vector $\widetilde{{\textbf{m}}}$ is drawn from the rows of the mask matrix $M$ and was applied to the input of $h$ such that for individual *i*, the *j*th phenotype would be masked when ${\widetilde{{{m}}}}_{j}=0$ or unmodified when ${\widetilde{{{m}}}}_{j}=1$. We controlled the prevalence of masking in training by the parameter $\rho$, which was the probability one individual would receive a copy-mask. The masking process of AutoComplete is illustrated in Fig. 1.

A joint loss function was defined over observed and masked values such that mean square error and cross entropy loss were applied to continuous and binary phenotypes respectively. For simplicity the two types of phenotypes were partitioned by index *C*. The joint loss function was applied over all values that were originally observed:

$${\textbf{y}}_{i,:}=h\left(\widetilde{{\textbf{m}}}\cdot {\widetilde{{\textbf{X}}}}_{i,:}\right)$$

$${L}_{i}\left(\varTheta \right)=\mathop{\sum }\limits_{j=1}^{C}{M}_{{ij}}{\left({y}_{{ij}}-{\widetilde{{\textbf{X}}}}_{{ij}}\right)}^{2}-\mathop{\sum }\limits_{j=C+1}^{P}{M}_{{ij}}\,\left[{\widetilde{{\textbf{X}}}}_{{ij}}\log \left({y}_{{ij}}\right)+\left(1-{\widetilde{{\textbf{X}}}}_{{ij}}\right)\log \left(1-{y}_{{ij}}\right)\right]$$

$$L\left(\varTheta \right)=\sum _{i}{L}_{i}\left(\varTheta \right)$$

The parameters $\varTheta \equiv \{{W}^{\,(1)},{{\textbf{b}}}^{(1)},{W}^{\,(2)},{{\textbf{b}}}^{(2)}\}$ of $h$ were optimized with respect to the objective $L$. Stochastic Gradient Descent^38^ was used to fit the neural net, where the initial learning rate, momentum and mini-batch size were also determined on a validation split of each dataset. The weights and biases of the network were initialized using the Kaiming Uniform distribution, and the slope parameter of LeakyReLU was initialized as ${l}_{\phi }=0.01$. Training proceeded given a maximum number of allowed epochs, up to 500, whereas the network weights were checkpointed based on a validation split which was randomly sampled from the training set to avoid overfitting. After training, the last checkpointed weights that attained the best validation loss were loaded back to the model for all imputation and downstream analysis. In Supplementary Fig. 2, we visualize the loss history recorded while fitting on the UKBB datasets. A single RTX8000 GPU was used to accelerate the fitting process of AutoComplete.

### Copy-masking

We implemented copy-masking, a simulation procedure to induce realistic patterns of missingness on observed data. This procedure was first used to simulate artificial missing data in the training and test splits of the datasets in the range of 1–50% for the purpose of assessing accuracy with structured missingness. For AutoComplete, we applied the same masking procedure as augmentations during training on top of the missing values already present with probability $\rho$ for a given individual. This approach strives to maintain the realistic missingness patterns in datasets while introducing simulated missing values. By contrast, uniform randomly withholding observed values could distort the distribution of the features; for example, when two features have correlated missingness. To illustrate the impact of copy-masking for imputation, we describe in Supplementary Note Section S6 the effect of using uniform masking for imputation performance in place of copy-masking and observe that no amount of uniform random masking alone attains the accuracy obtained with copy-masking (Supplementary Fig. 5).

### Hyperparameter tuning

For our simulation results, all methods were tested after tuning their hyperparameters on a validation dataset. For AutoComplete, HI-VAE and GAIN, we used the same predetermined portion (20%) of the samples not part of the test set as a validation set on which we evaluated hyperparameters after training on the remaining portion. SoftImpute was tuned using a *k*-fold (*k* = 5) cross-validation. For the AutoComplete final imputation results, we carried over the same hyperparameters which were found to be optimal in simulations.

In summary, the final set of notable hyperparameters chosen for AutoComplete were learning_rate = 0.1, copy_mask = 80%, batch_size = 2,048 and max_epochs = 500 for the psychiatric disorders dataset; and copy_mask = 30% for the cardiometabolic dataset. The copy-mask percentage was the main contributor to optimal accuracy, and other hyperparameters such as the momentum for Stochastic Gradient Descent optimization, learning rate decay of the scheduler and Leaky ReLU parameters were left fixed. For HI-VAE, the final set of hyperparameters chosen were *y* = 5, *z* = 16, *s* = 1, batch_size = 4,096 and max_epochs = 100. For GAIN, the final set of hyperparameters chosen were hint = 0.9, alpha = 10, batch_size = 4,096 and max_epochs = 2,000. To tune SoftImpute, we followed a cross-validation procedure as used previously^39^, where we chose a nuclear norm (Lambda) value of 108. Because of the difficulty in KNN and missForest scaling to the size of the cardiometabolic and psychiatric disorders dataset, we did not perform hyperparameter tuning for these methods (which would require repeated fits and evaluations). Reasonable values for hyperparameters were chosen instead. For KNN, the number of neighbors K was set to 10. For missForest, the number of trees per forest was set to 10 and up to 10 epochs were run. We did not alter hyperparameters that were not modifiable given each method’s software package. Supplementary Note Section S2 describes details on the specific hyperparameters that were tuned for each method.

### Details of GWAS analysis

We used imputed genotypes available from the UKBB for the individuals that were included in the phenotype imputation. We performed stringent filtering on the imputed variants, removing all insertions and deletions and multiallelic SNPs: we hard-called genotypes from imputed dosages at 9,720,420 biallelic SNPs with imputation INFO score >0.9, MAF >0.1% and *P* value for violation of Hardy–Weinberg equilibrium $> {10}^{-6}$, in individuals with a genotype probability threshold of 0.9 (individuals with genotype probabilities below 0.9 would be assigned a missing genotype). Of these, 5,776,313 SNPs are common (minor allele frequencies (MAF) >5%). We consistently use these SNPs for all analyses in this study.

We used 20 principal components (PCs) computed with FlashPCA^40^ on 337,126 white British individuals in UKBB and genotyping arrays as covariates for all GWAS. We performed principal component analysis on directly genotyped SNPs from samples in UKBB and used PCs as covariates in all our analyses to control for population structure. From the array genotype data, we first removed all samples that did not pass quality control, leaving 337,126 white British, unrelated samples. We then removed SNPs not included in the phasing and imputation and retained those with MAF ≥0.1%, and *P* value for violation of Hardy–Weinberg equilibrium $> {10}^{-6}$, leaving 593,300 SNPs. We then removed 20,567 SNPs that are in known structural variants and the major histocompatibility complex, as recommended by UKBB^16^, leaving 572,733 SNPs. Of these, 334,702 are common (MAF >5%), and from these common SNPs we further filtered based on missingness <0.002 and pairwise LD ${r}^{2} < 0.1$ with SNPs in a sliding window of 1,000 SNPs to obtain 68,619 LD-pruned SNPs for computing PCs using FlashPCA. We obtained 20 PCs, their eigenvalues, loadings and variance explained, and consistently use these PCs as covariates for all our genetic analyses.

The number of loci were counted from the GWAS results through a chromosome-wide clumping procedure. The top significantly detected SNP from one chromosome was tallied as a hit, and then all significant hits within 1 Mb from the SNP were ignored. The procedure was repeated for any remaining significant detection in the chromosome, and then repeated within all chromosomes.

### GWAS on AutoComplete-imputed phenotypes

For the imputation of phenotypes for which we performed GWAS, AutoComplete was allowed to fit all available individuals to impute missing entries. For binary phenotypes, phenotypes were imputed in a continuous range of 0–1 reflective of confidence in the prediction. When fitting all individuals, optimal hyperparameters were carried over from the tuning result of 1% missing data simulation. Similar to the simulation phase, during the final imputation procedure a portion of all samples were reserved as a validation set (20% by default), which was used to monitor for overfitting and perform weight saving. Therefore, all individuals present in the dataset were considered for the final imputation, and the sample size for downstream analyses was the total number of individuals in each dataset.

GWAS on originally observed UKBB phenotypes were performed with imputed genotype data at the 5,776,313 SNPs (MAF >5%, INFO score >0.9) using logistic regression or linear regression based on the data type of the phenotype (PLINK v.2)^41^. For all GWAS involving imputed phenotypes, linear regression was performed. We tally the number of significantly associated loci using the combination of observed and imputed individuals (all available individuals) and visualize their corresponding quantile-quantile plots in Supplementary Fig. 3.

### External GWAS datasets

We compared the GWAS on AutoComplete-imputed phenotypes with four GWAS results on external datasets. Direct bilirubin levels (field 30660) were measured for 226,876 unrelated white British individuals in the UKBB (58,531 missing). Imputed direct bilirubin was compared with measurements of bilirubin levels on 66,732 individuals from the Vanderbilt University Medical Center (VUMC) EHR system^25^. Diagnosis of LifetimeMDD^17^ for 67,165 individuals (269,963 missing) in the UKBB was validated against a comprehensive study of MDD across of 124,065 individuals by the PGC (excluding UKBB and 23andMe)^26^ and a study of 307,354 individuals carried out using data from 23andMe^17^. Finally, comparisons were made between the cannabis ever taken status in the UKBB (field 20453) for 110,189 individuals (226,939 missing) and a study of lifetime cannabis use across 32,330 individuals of European ancestry by the ICC^27^.

### Accounting for imputation uncertainty in downstream genomic analysis

We implemented a procedure involving multiple imputations through bootstrap resampling to account for uncertainty arising from imputation. This approach was applied to account for imputation uncertainty in downstream analyses such as when testing for genetic associations and measuring genetic correlations.

For a given dataset, we repeated the imputation procedure ten times using AutoComplete, which was fitted from scratch to reflect variations in imputation. Although the fitting procedure and hyperparameters were kept the same, the seed of the random generation was altered such that the weights would be initialized differently, mini batches would be formed in a differently shuffled order and the sequence of individuals randomly selected to receive copy-masking would change. In addition, we introduced bootstrapping to the fitting process such that the model was fit on a bootstrapped dataset in which all individuals were sampled with replacement, while the fitted model was used to impute the original dataset. This bootstrapping procedure accounts for the variation in the imputation model due to variation in the training samples (reflected in differences in the bootstrap samples), missingness patterns encountered (since copy-masking is applied independently in each bootstrapped sample), and to dependence on random parameter initialization.

We applied Rubin’s rule^3^ to utilize the multiple imputed datasets to account for imputation uncertainty in a downstream statistic. In the context of GWAS, an association study was performed for each imputation such that multiple effect size estimates and their standard errors were estimated per SNP. The significance of each SNP was determined by combining the point estimates and standard errors. Tallies of significantly associated loci in our results involving imputed phenotypes were based on this procedure. For genetic correlation analyses, the ${r}_{G}$ was measured between a nonimputation-based GWAS (UKBB or nonUKBB) and multiple imputation-based GWAS, and their statistics were combined while accounting for imputation uncertainty. Empirical observations on the change in the statistics due to imputation are further described in Supplementary Note Section S4.

### Additional analysis of imputed phenotypes

The effective sample size was calculated as a function of imputation accuracy for a given phenotype from simulations (1% missingness) and the number of missing values imputed, such that ${N}_{{{\textrm{Effective}}}}={N}_{{{\textrm{Observed}}}}+{r}_{{{\textrm{AutoComplete}}}}^{2}\times {{N}}_{{{\textrm{Imputed}}}}$ for a given phenotype.

We examined genetic correlations (${r}_{G}$) between a subset of phenotypes within the psychiatric disorder dataset collected within the UK Biobank and related phenotypes collected from cohorts outside the UK Biobank. The three phenotypes examined based on the UK Biobank were direct bilirubin, LifetimeMDD^17^ and status of having ever taken cannabis. In the context of these phenotypes, we gathered GWAS summary statistics from external studies that examined bilirubin measurements^25^, MDD^17,26^ and lifetime cannabis use^27^. We used LDSC^28^ to estimate ${r}_{G}$ between each pairing of phenotypes using LD Scores estimated from the 1,000 Genomes white European population^42,43^.

### Reporting summary

Further information on research design is available in the Nature Portfolio Reporting Summary linked to this article.

## Online content

Any methods, additional references, Nature Portfolio reporting summaries, source data, extended data, supplementary information, acknowledgements, peer review information; details of author contributions and competing interests; and statements of data and code availability are available at 10.1038/s41588-023-01558-w.

### Supplementary information

Supplementary Information Supplementary Note, Figs. 1–6 and Tables 1–5.

Reporting Summary

## Data Availability

The genotype and phenotype data are available by application from the UKBB, https://www.ukbiobank.ac.uk. The LD Scores from the 1000 Genomes project are available from https://alkesgroup.broadinstitute.org/LDSCORE/. Further data are available as follows: Bilirubin GWAS^25^, http://ftp.ebi.ac.uk/pub/databases/gwas/summary_statistics/GCST90012001-GCST90013000/GCST90012749/; MDD GWAS by PGC (excluding UKBB and 23andMe)^26^, https://figshare.com/articles/dataset/mdd2018/14672085; MDD GWAS of 23andMe cohort^17^, https://figshare.com/s/b61e44d5142cc0690772; Lifetime cannabis use GWAS^27^, https://www.ru.nl/bsi/research/group-pages/substance-use-addiction-food-saf/vm-saf/genetics/international-cannabis-consortium-icc/. The following GWAS of phenotypes after imputing all missing entries are available from the GWAS Catalog with the accession codes: bilirubin, GCST90277451; cannabis ever taken, GCST90277452; and LifetimeMDD, GCST90277450.

## Code availability

The software can be accessed as follows: AutoComplete, https://github.com/sriramlab/AutoComplete (10.5281/zenodo.8243106); Plink 2.0, https://www.cog-genomics.org/plink/2.0/; LDSC, https://github.com/bulik/ldsc; HI-VAE, https://github.com/probabilistic-learning/HI-VAE; GAIN, https://github.com/jsyoon0823/GAIN; KNN, https://scikit-learn.org/stable/modules/generated/sklearn.impute.KNNImputer.html; MissForest, https://cran.r-project.org/web/packages/missForest/index.html; MICE, https://github.com/AnotherSamWilson/miceforest.
