# Supplementary material for: Deep learning-based phenotype imputation on population-scale biobank data increases genetic discoveries
Source: Nat Genet. 2023 Nov 20;55(12):2269–76. doi: 10.1038/s41588-023-01558-w (PMC10703681; doi:10.1038/s41588-023-01558-w)
Supplement: Supplementary file 1 — Supplementary Note, Figs. 1–6 and Tables 1–5. [file 41588_2023_1558_MOESM1_ESM.pdf]

nature genetics

Technical Report https://doi.org/10.1038/s41588-023-01558-w

Deep learning-based phenotype imputation on population-scale biobank data increases genetic discoveries

In the format provided by the authors and unedited

Supplementary Note: Deep Learning-based Multiple Phenotype Imputation on Biobank-scale Data Increases Genetic Discoveries

S1 Related Work

When a subset of features are missing consistently, the missing values can be predicted using the remaining observed features using standard supervised learning. In practice, patterns of missingness can be complex. Multivariate imputation by chained equations (MICE) [1], which aims to repeatedly fit a conditional distribution for each feature given the other and using this distribution to impute missing values, has emerged as a principled framework to dealing with missing data. Several variants of this approach, based on how the conditional distributions are modeled, have been developed and methods which leverage random forests within MICE such as MissForest [2] and MICE-Forest [3] are widely used. These approaches have the advantage of being able to handle mixed data types. K-Nearest Neighbors [4] is also a prevalent approach for imputation of continuous data (originally introduced to impute gene expression data). This approach attempts to impute a missing phenotype based on K "nearest" phenotypes (determined by computing a distance on the observed phenotypes). The accuracy of this approach depends on the choice of K and appropriate choice of a measure of distance across phenotypes [5].

Approaches such as the Multivariate Normal Model [6], MissGLasso [7], MissPALasso [8], and TRCMA [9] aim to estimate the parameters of the distribution of the data, in some cases with additional regularization. MissGLImp and MissPALasso aim to learn a sparse inverse-covariance matrix underlying the partially observed phenotypes using a EM-style algorithm while TRCMA aims to fit a matrix-normal model to the matrix of phenotypes over individuals (with some entries missing) where the covariance matrices underlying the matrix normal model are regularized. SoftImpute [10] has become one of the most prevalent imputation methods due to its scalability and flexibility [11,12]. The method builds upon work in matrix completion, including SVD-based imputation [13] and HardImpute [14]. It assumes that the latent phenotype matrix has low-rank that it attempts to estimate by searching for a matrix that is close to the observed entries of the phenotype matrix while also having an approximately low rank (as quantified by its nuclear norm).

In the context of genetic studies, PHENIX [15] models the matrix of phenotypes observed across individuals (with some entries missing) as arising from a matrix-normal distribution. This elegant approach accounts for the genetic relatedness (that can induce correlations for a given phenotype measured across individuals) and pleiotropy (that can induce correlations across phenotypes measured in the same individual). The parameters of the model are estimated using a variational Bayes algorithm which also provides an approximate posterior distribution over the missing phenotypes. While an elegant model, it is challenging to scale PHENIX to large numbers of individuals and phenotypes (for

example, this would require eigendecomposition of the kinship or genetic relatedness matrix). Further, the model underlying PHENIX is designed for normally-distributed phenotypes (the method was shown to be less accurate on non-normally distributed phenotypes though it remained the most accurate compared to other methods) [15].

PhenIMP [16] considers the setting where phenotypes that are closely related to a phenotype of interest have been collected in large samples. PhenIMP models the joint distribution of the phenotype of interest and related phenotypes in each individual as arising independently from a zero-mean multivariate normal distribution. The covariance matrix of the distribution over the phenotypes is estimated from a dataset where all phenotypes are measured which can then be used to impute the phenotype of interest in datasets where only the related phenotypes are measured. PhenIMP requires access to a complete dataset on which the phenotype of interest and related phenotypes are measured and is also designed for normally distributed phenotypes.

Recent advancements in deep-learning [17] have given rise to deep generative models which are capable of learning high-dimensional, multi-modal distributions. Notably, Variational Auto-Encoders (VAE) offer one framework of using Deep Neural Nets (DNN) to build a probabilistic model [18]. Variants of VAE have been proposed for the imputation problem [19] though the proposed approach assumes the presence of samples with complete observations to learn the model parameters. HI-VAE [20] defines a comprehensive deep probabilistic model which accounts for missingness in observations, as generated from a mixture distribution, and natively supports various heterogeneous data formats (continuous, binary, categorical, ordinal). The flexibility of the model makes it one of the most suitable deep-learning methods in the context of medical data which may consist of all the noted attributes. In addition to VAEs, Generative Adversarial Networks (GAN) [21] offer an alternative approach to generative modeling using a deep generator-discriminator paired architecture. GAIN [22] extends GANs to the imputation problem and has been shown to obtain improved accuracy over MICE and MissForest [1,2,23]. However HI-VAE has been shown to be favorable in comparison to GAIN under varying datasets [20] and the lack of consistency in the convergence of GANs has been one barrier to their broader use [24].

Among deep-learning methods, Auto Encoders (AE) have remained a competitive approach for imputation. The method is based on discriminative training of deep encoder-decoder neural nets with a focus on reconstructing perturbed or missing values. Several works have used AEs for imputing medical records. The earlier works, however, relied on mostly complete datasets with high proportion of observed values, such that underlying missingness was not a consideration for the methods, and their utility was mostly demonstrated for synthetically created levels of missing data. Beaulieu-Jones et al. [25] demonstrated that medical records relating to ALS [26] could be imputed with higher accuracy using AE than several non deep-learning methods. However, the work was defined specifically for one data format (binary labels), and evaluated on a synthetic dataset of missing values generated from 2000 individuals. DeepImpute [27] similarly demonstrated the applicability of AEs in on single-cell RNA-seq data where completely observed samples were available, evaluating on incomplete datasets which were simulated.

Phung et al. [12] proposed a denoising auto-encoder [28,29] where Gaussian noise was dynamically added to the data, successfully improving imputation for infant mortality records. DeepPatient [30] leveraged stacked DAEs under uniform masking noise to fit medical records. While not explicitly defined for imputation, this work demonstrated that the latent representations learned by DAEs were predictive of various diseases.

Novel deep neural-net architectures are continually developed such as attention-based [31], graph-based [32], or causally regularized [33] methods, but several aspects of AutoEncoders, the effectiveness of denoising, and their extension to highly missing, massive datasets for real-world impact have yet to be explored. Our work emphasizes the strong performance and reliability of DAEs, and we arrive at an imputing DAE which is favorable to many conventional and deep-learning approaches which generalizes to the types of missingness found in biobank-scale data.

S2 Hyperparameter Tuning and Fitting

A single Quadro RTX 8000 was used to accelerate learning for HI-VAE, GAIN, and AutoComplete which were implemented in PyTorch or Tensorflow enabling GPU usage. SoftImpute and K-Nearest Neighbors were fitted on a workstation equipped with the AMD EPYC 7501 32-Core 3GHz Processor and up to 1024 GB of RAM.

Hyperparameter tuning was performed for all methods using the same predetermined training split of each dataset for all methods. In Supplementary Table 3, the results of the hyperparameter search for each method and the final choices are outlined. For the deep learning methods (AutoComplete, HI-VAE, and GAIN), 80% of the samples were used to learn the parameters for fixed choices of hyperparameters and the remaining 20% was used to evaluate the given hyperparameter choice. For each method, we focused our tuning efforts on hyperparameters that lead to the biggest change in validation accuracy while leaving all other hyperparameters fixed. The final hyperparameter choice based on the tuning results were determined by imputation accuracy (r2) within the validation set.

AutoComplete: We tuned the percentage of samples which received copy-masking during training. Tuning the learning rate or the batch size did not lead to notable differences in the validation accuracy. The final set of hyperparameters chosen were { learning_rate=0.1, copy_mask=80%, batch_size=2048, max_epochs=500 }.

HI-VAE: We tuned the latent dimension size (z) from 2, 8, 16 and the dimension of the MLP assigned to each phenotype (y) from 1, 5, 10. The number of mixtures was fixed to 1 for all experiments.The largest possible batch size was chosen such that it could be handled by the GPU. Other than the number of epochs, no other hyperparameters were modifiable using the HI-VAE package. The final set of hyperparameters chosen were { y=5, z=16, batch_size=4096, max_epochs=100 }.

GAIN: We tuned the Hint fraction in the range of 0.1, 0.5, 0.9 which weights the amount the discriminator penalizes missing values imputed by the generator and the Alpha multiplier which weights the reconstruction loss in the objective relative to the discriminative loss in

the range of 0.001, 0.1, 1, 10, 20. Other than the number of epochs and batch size, no other hyperparameters were modifiable using the GAIN package. We did not observe notable changes to the model fit when adjusting batch size. The final set of hyperparameters chosen were { hint=0.9 , alpha=10, batch_size=4096, max_epochs=2000 }.

To tune SoftImpute, we followed a cross validation procedure as used in [34] where the nuclear norm penalty Lambda was tuned in a range starting with its largest possible value given the data (approximately 1289, as returned by the `lambda0` function in the SoftImpute package) and decreasing it over 20 intervals down to 1e-2 in log scale. The mean-squared error was used to assess the accuracy of the reconstructed training matrix. A Lambda value of 108 was chosen based on the reconstruction metric.

Due to the difficulty in K-Nearest Neighbors and MissForest scaling to the size of the Cardiometabolic and Psychiatric Disorders Dataset, we did not perform hyperparameter tuning for these methods (which would require repeated fits and evaluations). Reasonable values for hyperparameters were chosen instead. For K-Nearest Neighbors, the number of neighbors K was set to 10. For MissForest, the number of trees per forest was set to 10 and up to 10 epochs were run.

When fitting AutoComplete for tuning, simulations, and the final imputation, the network weights were checkpointed based on a validation split (20%), where the criterion was an improvement in the objective at every epoch. After training, the checkpointed weights for the best validation loss attained were loaded for imputation and used for all downstream tasks. We allowed training to continue for 500 epochs, while the best weights were saved before the maximum number of epochs were reached. In Supplementary Figure 2, we visualize the loss history recorded for the training regime which produced the final imputation networks for the Cardiometabolic and the Psychiatric Disorders dataset.

We did not observe significant amounts of overfitting when training AutoComplete that would alter our findings. For the Psychiatric Disorders dataset, the training, validation, and test losses at the last checkpoint were 0.326, 0.329, and 0.327. For the Cardiometabolic dataset, the training, validation, and test losses at the last checkpoint were 0.0573, 0.0575, and 0.0580.

S3 Evaluation of runtime

In Supplementary Figure 1, we illustrate the effect of training time to imputation accuracy for the main methods in our comparison. All methods were run to provide only one imputed matrix, as opposed to performing multiple imputation. We note that multiple imputations can generally be performed independently of one another, allowing parallelization which would roughly result in similar running times as a single run given sufficient compute resources. Specifically for AutoComplete, we note that our software package can generate a list of commands which may be run independently and in parallel to obtain multiple imputations.

We visualized the average r2 accuracy obtained according to the same amount of time (in minutes) elapsed during the model training procedure across comparable methods with a maximum allocation of 30 minutes for each method. For all methods which iteratively optimize their parameters, we imputed the data matrix using the partially optimized parameters at the end of each epoch and measured the phenotype-wise average r2. We visualized the r2 accuracies as a measure of imputation progress over the same amount of time budgeted for the compared methods. The accuracies were measured on the test split of the Psychiatric Disorders dataset (337,126 individuals and 372 phenotypes), where each method imputed a set of observed values set to be missing (1% simulated missingness). The choice of hyperparameters for all methods were fixed to the values carried over from the tuning performed in the 1% simulation setting. While each of KNN (using K=10), MissForest (1 tree per forest), and MICE required more than the maximum allotted time, we nevertheless reported the accuracy of each of these methods. KNN algorithm required 12 hours to impute this dataset. Similarly, we also reported the imputation accuracy of MissForest after 1 iteration (3 ½ hours) and MICE after 3 iterations (6 hours) to provide further context on their scalability.

Although hyperparameters and alternative initializations can lead to variation in total runtime of any method including AutoComplete, we observed that AutoComplete remains well-suited for practical use. SoftImpute, HI-VAE, and GAIN terminated before the 30 minutes allotted while attaining r2 accuracies which were lower than AutoComplete at the corresponding point in time. While the termination criterion may be adjusted, it appeared unlikely that the compared methods would continue to improve r2 accuracy. AutoComplete attained the highest r2 accuracy after 30 minutes and continued to improve r2 accuracy up to approximately ~1 hour where the weights were last checkpointed.

A single Quadro RTX 8000 was used to accelerate the training for HI-VAE, GAIN, and AutoComplete which were implemented in PyTorch or Tensorflow enabling GPU usage. SoftImpute and K-Nearest Neighbors were fitted on a workstation equipped with the AMD EPYC 7501 32-Core 3GHz Processor and up to 1024 GB of RAM.

S4 Change in genomic analysis after accounting for uncertainty

We implemented a bootstrapping procedure to produce 10 multiple imputations in order to account for imputation uncertainty in downstream genomic analysis. This bootstrapping procedure accounts for the variation in the imputation model due to variation in the training samples (reflected in differences in the bootstrap samples), missingness patterns encountered (since copy-masking is applied independently in each bootstrapped sample), and to dependence on random parameter initialization. To obtain GWAS SNP effect estimates that account for imputation uncertainty for each of the three phenotypes (Direct Bilirubin, LifetimeMDD, and Cannabis Ever Taken), the GWAS effect sizes across multiple imputed datasets were combined following Rubin's rule.

As expected, this process led to an increase in the standard errors (SEs) for the effect sizes across the SNPs (relative to analyzing a single imputation). The SEs for the effect sizes across all SNPs increased (mean and standard deviations of the standard errors): 0.00732 (0.00193) to 0.00733 (0.00193), 0.00364 (0.00096) to 0.00367 (0.00097), and 0.00170 (0.00045) to 0.00373 (0.00099) for the three phenotypes respectively. Among significantly associated loci, SEs increased: 0.00802 (0.00216) to 0.00807 (0.00217), 0.00320 (0.00076) to 0.00323 (0.00077), and 0.00152 (0.00026) to 0.00334 (0.00059) respectively.

S5 Tests in Smaller Scales

We organized a smaller subset of 86 phenotypes related to blood lab measurements within the cardiometabolic dataset called the Blood Labs dataset to test a wider variety of methods which could not scale to the size of the two main datasets to either perform hyperparameter tuning effectively or run inference until convergence (K-Nearest Neighbors, MissForest). This dataset contained 68 continuous and 18 binary phenotypes, with the phenotype with highest missingness being 91% missing. We randomly selected 100,000 individuals (out of a total of 291,273) as a smaller subset of which 50,000 individuals were used to train or tune the imputation methods and testing was performed on the remaining 50,000. In this setting, we could perform hyperparameter search and fitting to the fullest extent for three widely applied methods, KNN [4], MissForest [2], and MICE [1] in reasonable time. Based on a further validation split (20% of the training split), the optimal hyperparameters chosen for KNN was k=60 nearest neighbors and up to 80 trees for MissForest. MissForest was configured to allow fitting up to 100 maximum iterations. We use a random forest-based implementation of MICE [1] which performed hyperparameter tuning internally, and was configured to fit for 10 iterations and use the point estimate of 5 multiple imputations as the final imputed values.

On this small-scale dataset, the accuracy of each of the methods declined with increasing missingness as seen on the main datasets (Supplementary Figure 4.a). Given reduced sample sizes, the confidence intervals were observed to be larger than results from the two main datasets. Nevertheless, AutoComplete and SoftImpute appeared as the top two most accurate methods. While the methods were comparable for the 1% simulated setting (AutoComplete improved 1 phenotype with significance and none decreased with significance), AutoComplete was found to improve a notable number of phenotypes for greater simulated missingness (13 improved and 4 decreased with significance for 20%, Supplementary Figure 4.b).

On average across all simulations (1%~50%) AutoComplete was observed to be favorable in comparison to MissForest, increasing r2 from 0.180 (0.003) to 0.202 (0.003) was observed (+12%), and in comparison to KNN, increasing r2 from 0.159 (0.004) to to 0.202 (0.003) (+27%). We also evaluated the accuracy of AutoComplete which could be trained on all training data (N=151,273) before imputing the smaller setting (“AutoComplete (full)”) .

This approach was observed to obtain the highest accuracy among the approaches compared.

S6 Contribution of copy-masking

We performed ablation tests to determine that copy-masking is a key factor in improving imputation accuracy for AutoComplete. We measured average r^2 across phenotypes from the psychiatric disorders dataset with increasing percentages of simulated missingness ($1\% \sim 50\%$ missing). We compared AutoComplete with training the denoising autoencoder with uniform random masking of observed values in increasing amounts of $10\% \sim 90\%$ (Supplementary Figure 5). For the simulated setting of 1% missingness, the highest average r^2 obtained through uniform masking was 0.128 in comparison to 0.143 with AutoComplete (12% improvement) with similar trends in tests with increasing missingness (19% improvement average across simulations). We therefore conclude that AutoComplete benefits substantially from being trained on realistic missingness patterns that aid the denoising behavior of the deep learning model.

Additionally, we tested the effect of not using copy-masking in terms of the evaluation of the imputation methods, allowing the imputation methods to leverage any trivial correlations that exist between phenotypes but do not reflect the observed patterns of missingness. To test this setting, we simulated missingness uniformly and independently across phenotypes as a percentage of the total observed values from 1% to 50%, disregarding any underlying masking patterns observed in the data. We observe that copy-masking led to a substantially higher imputation accuracy for the psychiatric disorders phenotypes, where for the 1% missing setting the r^2 of LifetimeMDD imputed using AutoComplete increased from 0.507 in copy-masked evaluations to 0.968 (nearly 100%) in uniform masked evaluations (Supplementary Figure 6).

Supplementary Tables and Figures

Dataset	N	# Pheno.	# Cont.	# Binary	$\varnothing_N$	$\varnothing_P$
Cardiometabolic	285,405	230 (49%)	46 (7%)	184 (60%)	50% (78%)	47% (99%)
Psychiatric Disorders	337,126	372 (46%)	98 (55%)	274 (42%)	53% (77%)	67% (99%)

Supplementary Table 1: We collected two sets of phenotypes from two studies related to the UK Biobank. Each dataset contains hundreds of thousands of individuals (*N*) and a heterogeneous mix of continuous (*Cont.*) and binary valued phenotypes. Percent of all values which are missing for select phenotypes are reported in parentheses after the number (#) of such traits. We report the median percentage of measurements missing per individual ($\varnothing_N$) (maximum missing in parentheses) in addition to the median percentage of measurements missing per phenotype ($\varnothing_P$).

		Cardiometabolic					Psychiatric Disorders				
	Method	1%	5%	10%	20%	50%	1%	5%	10%	20%	50%
r^2	SI	20(16)	56(40)	73(51)	85(42)	158(3)	38(1)	114(7)	159(13)	179(19)	206(0)
	HIVAE	92(6)	123(11)	139(11)	159(6)	178(3)	68(0)	126(1)	166(1)	181(2)	208(0)
	KNN	123(4)	163(4)	171(4)	177(4)	183(0)	108(0)	169(0)	194(0)	212(0)	207(0)
	GAIN	58(6)	132(9)	150(10)	170(8)	180(4)	130(0)	180(0)	203(0)	220(0)	198(0)
AUPR	SI	3(0)	4(0)	6(0)	6(0)	9(0)	7(0)	38(1)	78(5)	108(15)	115(2)
	HIVAE	4(0)	5(0)	5(0)	6(0)	12(0)	20(0)	54(0)	88(0)	101(0)	126(0)
	KNN	4(0)	6(0)	9(0)	10(0)	12(0)	42(0)	91(0)	111(0)	123(0)	116(31)
	GAIN	4(0)	9(0)	9(0)	12(0)	13(0)	72(1)	109(5)	127(6)	133(7)	131(11)
AUROC	SI	3(0)	5(0)	6(0)	6(1)	10(0)	13(0)	67(1)	98(9)	112(19)	128(0)
	HIVAE	4(0)	5(0)	6(0)	6(0)	12(0)	31(0)	72(0)	107(0)	114(0)	137(0)
	KNN	5(0)	7(0)	9(0)	13(0)	13(0)	78(0)	122(0)	130(0)	141(0)	151(0)
	GAIN	5(0)	10(0)	8(0)	12(0)	13(0)	94(0)	132(0)	146(0)	153(0)	148(0)

Supplementary Table 2: Number of phenotypes for which AutoComplete significantly improved imputation accuracy relative to other methods. Significant decreases in accuracy are indicated in parentheses. Imputation accuracy was determined through a simulation of 1%~50% missingness on the cardiometabolic and psychiatric disorders datasets, for the metrics of squared Pearson correlation (r^2), Area Under Precision Recall (AUPR), and Area Under Receiver Operating Characteristic (AUROC).

AutoComplete	
Copymask %	r2
0	0.123
10	0.135
50	0.153
**80**	**0.157**
90	0.155

SoftImpute	
Lambda	MSE
1289	0.069
694	0.063
374	0.057
201	0.053
**108**	**0.052**
58	0.054
31	0.058

HI-VAE			
	z		
y	2	8	**16**
1	0.0387	0.0414	0.0429
**5**	0.0318	0.0387	**0.0429**
10	0.0425	0.0356	0.0388

GAIN			
	Hint		
Alpha	0.1	0.5	**0.9**
20	0.0073	0.0049	0.00759
**10**	0.00239	0.00327	**0.00827**
1	0.00209	0.0025	0.00357
0.1	0.00461	0.00254	0.00266
0.001	0.00232	0.00232	0.00356

Supplementary Table 3: Results of hyperparameter tuning of all methods. The results of hyperparameter tuning of the main hyperparameters for each method. AutoComplete was tuned primarily based on the copy-masking percentage. HI-VAE was tuned based on the size of the latent dimension (z) and size of the MLP per phenotype (y). GAIN was tuned based on the Hint fraction and Alpha multiplier. SoftImpute was tuned based on the nuclear norm penalty Lambda in a range starting from a value preferring optimal reconstruction of observed values (1289, as returned by the `lambda0` function in the SoftImpute package) down to 1e-2 in 20 steps in the log scale (first 7 steps shown). Bold denotes the final hyperparameter values were chosen to maximize r2 on the validation dataset (20% of the training set that was not used for learning).

Phenotype	N	r^2	N Imputed	N Effective	Increase
Bilirubin	226,897	0.51	58,597	256,692	13%
LifetimeMDD	67,164	0.51	269,962	193,379	187%
Cannabis Ever Taken	110,188	0.24	226,938	165,291	50%

Supplementary Table 4: Increase in effective sample sizes from AutoComplete imputations. The available sample size of each phenotype (N) was smaller than the number of total individuals in each dataset due to missingness. All missing measurements were imputed ($N_{Imputed}$) using AutoComplete. The accuracy of the imputation method for each phenotype could be measured in simulations (r^2), allowing an approximation of the hypothetical effective sample size ($N_{Effective} = N + r^2 \times N_{Imputed}$) and an estimate of the increase in sample size (Increase).

UKBB Imp-Obs			
Phenotype	Effect*	Effect	Sig
Bilirubin	17/17	17/17	17/17
LifetimeMDD	23/23	14/14	14/23
Cannabis Ever Taken	11/11	7/7	7/11

		Obs-Ext			Imp-Ext		
Phenotype	Study	Effect*	Effect	Sig	Effect*	Effect	Sig
Bilirubin	[72]	27/32	14/15	15/32	11/14	8/9	9/14
LifetimeMDD	[73]	1/1	0/0	0/1	18/18	13/13	13/18
LifetimeMDD	[51]	1/1	0/0	0/1	20/20	16/16	16/20
Cannabis Ever Taken	[74]	0/0	0/0	0/0	9/9	1/1	1/9

Supplementary Table 5: The significantly detected loci for imputed phenotypes in the UKBB (Imp) was compared with their observed counterpart in the UKBB (Obs) and external studies (Ext). Counts were tallied in terms of how many loci match in effect direction of the SNP (Effect), where effect size was distinct from zero ($p < 0.05$), and how many were marginally significant overall denoted as Sig ($p<0.05$). Significances were determined using a two-sided *t*-test. Also shown are the number of SNPs with matching effect direction regardless of it being significantly distinct from zero (Effect*). Denominator indicates the total number of SNPs present in the compared study and matching the criteria for comparison.

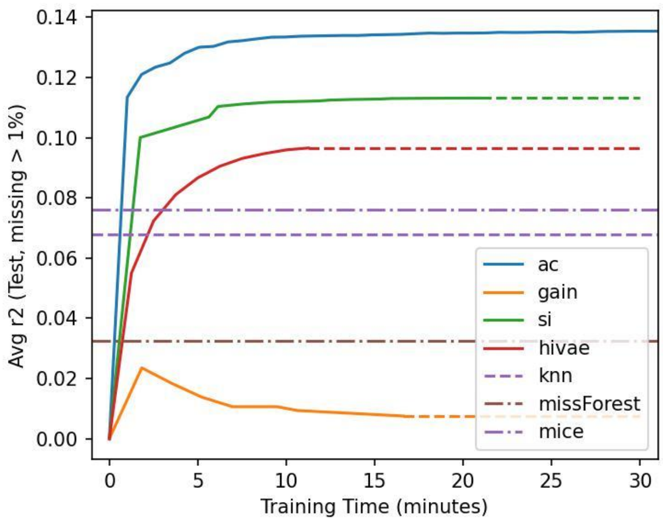

Supplementary Figure 1: Imputation accuracy (r2) as a function of the time allocated to train each method that uses iterative optimization (with a maximum of 30 minutes). Running time was measured for a single run of each method so that the runtime of AutoComplete does not include the time needed to run on multiple bootstraps. Each curve connects the r2 accuracy of the imputed data matrix on the test split of the Psychiatric Disorders dataset (337,126 individuals and 372 phenotypes; 1% simulated missing) given the partially optimized parameters fit up to the given time. Dotted lines indicate the final r2 accuracy reached by each method after terminating. The imputation accuracy of KNN (with K=10 neighbors), a non-parametric method that does not involve iterative optimization, is also visualized (horizontal dashed line) (finished in 12 hours). We also visualized the imputation accuracy of MissForest (with 1 tree per forest) after 1 iteration (3 1/2 hours) and MICE after 3 iterations (6 hours).

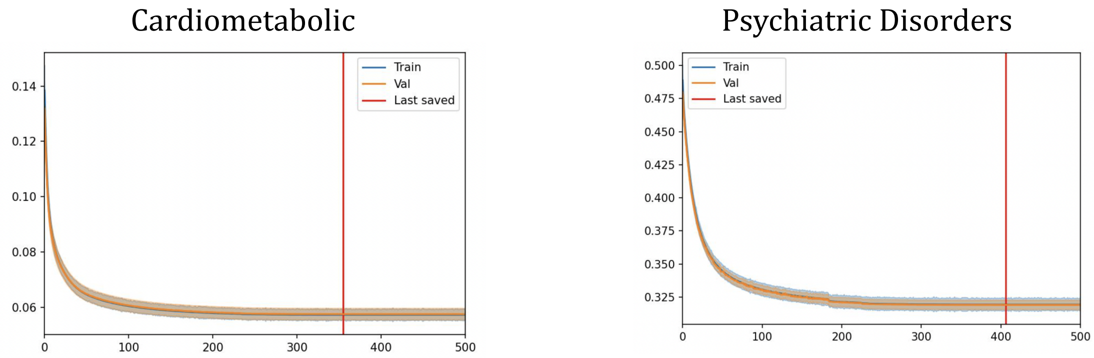

Supplementary Figure 2: The loss history while fitting AutoComplete on both Cardiometabolic and Psychiatric Disorders datasets on the training set (blue) and validation set (orange). Solid colored lines indicate mean loss with one epoch. 95th percentile of the loss during the epoch shaded for each phase. The point where validation

loss no longer improved (the optimized weights were no longer checkpointed) is indicated as “Last saved”.

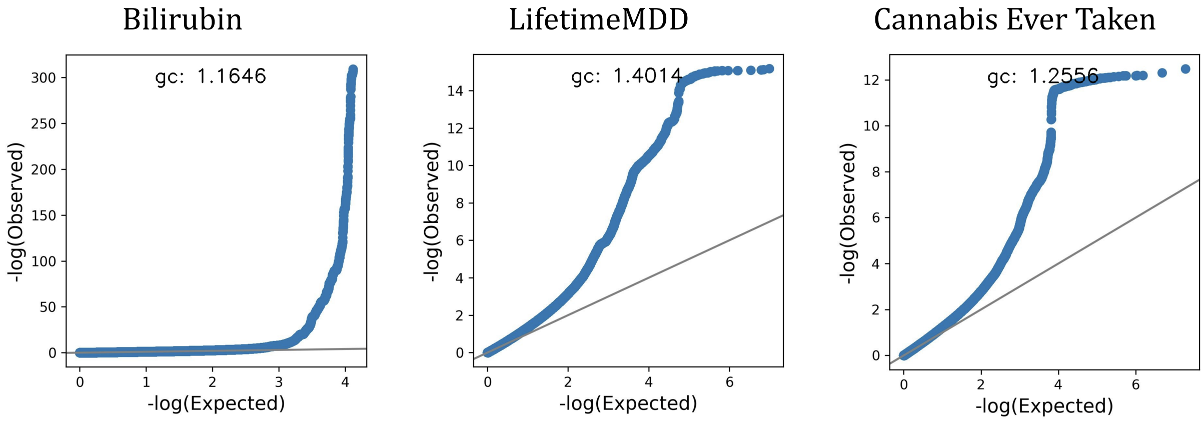

Supplementary Figure 3: QQ-plots corresponding to GWAS of three validated phenotypes after imputation with AutoComplete.

(a)

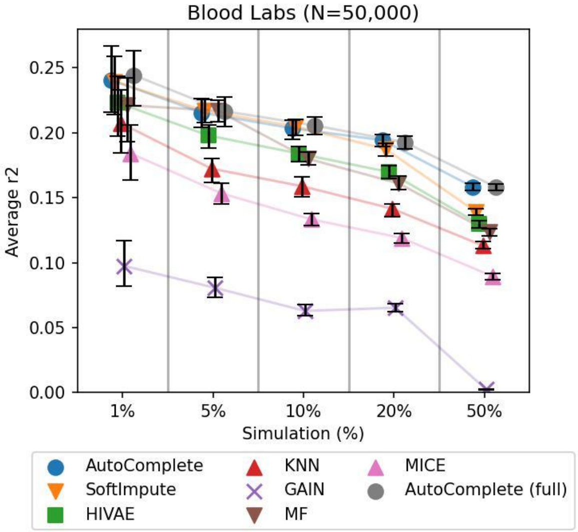

(b)

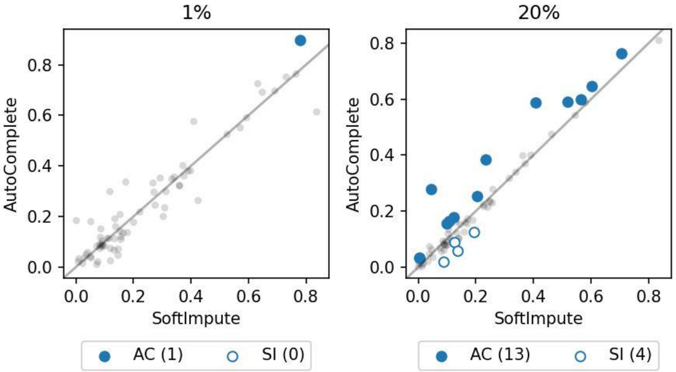

Supplementary Figure 4: (a) Imputation accuracy (r^2) was evaluated for a smaller setting of N=50,000 randomly drawn from a dataset of 86 phenotypes related to blood labs for which KNN, MissForest (MF), and MICE could be effectively fit (black bars denote 95% CIs, 100 bootstrap replicates). All methods were trained or tuned on an identically sized training set of 50,000 individuals (distinct from the 50,000 individuals used for evaluating accuracy). Accuracy obtained from fitting AutoComplete on the full training set of N=151,273 then imputing the smaller setting is also shown ("AutoComplete (full)"). **(b)** Imputation accuracy measured for each phenotype was compared between AutoComplete and SoftImpute (next best) for settings of 1% and 20% missing data. Accuracies significantly greater for AutoComplete are marked with filled bold dots and those which are significantly greater for the alternate method are marked with empty bold dots (two-sided t-test with $p < 5.81 \times 10^{-4}$, adjusted for the number of phenotypes).

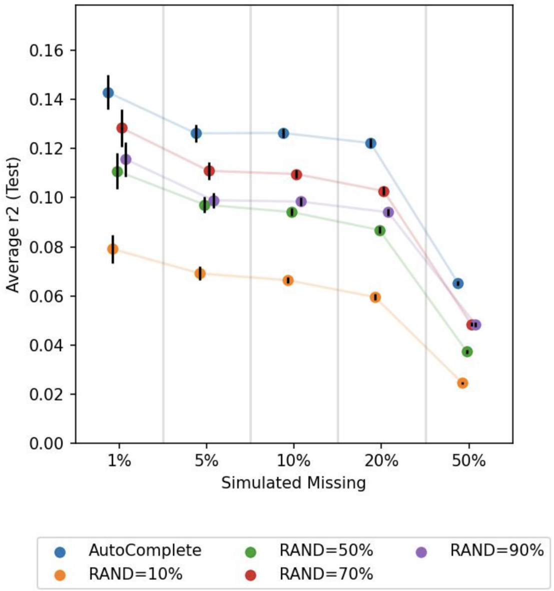

Supplementary Figure 5: Comparison of r2 in simulations of 1%~50% missing data on the Psychiatric Disorders dataset for different masking strategies (black bars denote 95% CIs, 100 bootstrap replicates). AutoEncoders were fitted under increasing probabilities of uniformly random masking (RAND=10%~90%) and compared with the final AutoComplete model which was fitted using copy masking (Psychiatric Disorders dataset: 372 phenotypes and 337,126 individuals).

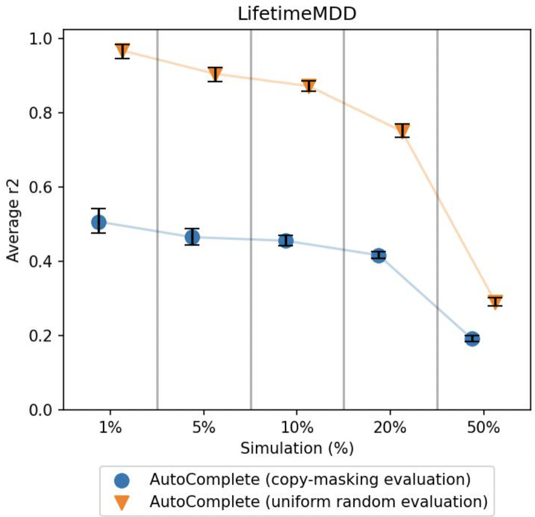

Supplementary Figure 6: Evaluation of imputation accuracy with and without copy-masking for LifetimeMDD (black bars denote 95%, 100 bootstrap replicates). Observations were chosen uniformly and independently across phenotypes to be scored instead of reflecting any masking patterns ("uniform random evaluation") and compared to our standard evaluation which uses copy-masked values. The AutoComplete model which

was tuned for the main experiments was reused for this setting. Accuracies are reported across increasing percentages of simulated missingness of 1%~50% (Psychiatric Disorders dataset: 372 phenotypes and 337,126 individuals).

References

1. van Buuren S. Flexible imputation of missing data. Second edition. Boca Raton, FL.: CRC Press; 2018.

2. Stekhoven DJ, Bühlmann P. MissForest—non-parametric missing value imputation for mixed-type data. Bioinformatics. 2011 Oct;28(1):112–8. Available from: https://doi.org/10.1093/bioinformatics/btr597

3. van Buuren S, Oudshoorn K. Flexible multivariate imputation by MICE. TNO; 1999.

4. Troyanskaya O, Cantor M, Sherlock G, Brown P, Hastie T, Tibshirani R, et al. Missing value estimation methods for DNA microarrays . Bioinformatics . 2001 Jun;17(6):520–5. Available from: https://doi.org/10.1093/bioinformatics/17.6.520

5. Beretta L, Santaniello A. Nearest neighbor imputation algorithms: A critical evaluation. BMC Medical Informatics and Decision Making. 2016 Jul 25;16(3):74. Available from: https://doi.org/10.1186/s12911-016-0318-z

6. Little RJ, Rubin DB. Statistical analysis with missing data. Vol. 793. John Wiley & Sons; 2019.

7. Städler N, Bühlmann P. Missing values: Sparse inverse covariance estimation and an extension to sparse regression. Statistics and Computing. 2012 Jan;22(1):219–35. Available from: https://doi.org/10.1007/s11222-010-9219-7

8. Städler N, Stekhoven DJ, Bühlmann P. Pattern alternating maximization algorithm for missing data in high-dimensional problems. J Mach Learn Res. 2014 Jan;15(1):1903–28.

9. Allen GI, Tibshirani R. Transposable regularized covariance models with an application to missing data imputation. The Annals of Applied Statistics. 2010;4(2):764–90. Available from: https://doi.org/10.1214/09-AOAS314

10. Hastie T, Mazumder R, Lee JD, Zadeh R. Matrix completion and low-rank SVD via fast alternating least squares. Journal of machine learning research : JMLR . 2015;16:3367–402. Available from: https://pubmed.ncbi.nlm.nih.gov/31130828

11. Mongia A, Sengupta D, Majumdar A. McImpute: Matrix completion based imputation for single cell RNA-seq data. Frontiers in Genetics. 2019;10:9. Available from: https://www.frontiersin.org/article/10.3389/fgene.2019.00009

12. Phung S, Kumar A, Kim J. A deep learning technique for imputing missing healthcare data. In: 2019 41st annual international conference of the IEEE engineering in medicine and biology society (EMBC). 2019. p. 6513–6.

13. Koren Y, Bell R, Volinsky C. Matrix factorization techniques for recommender systems. Computer. 2009;42(8):30–7.

14. Mazumder R, Hastie T, Tibshirani R. Spectral regularization algorithms for learning large incomplete matrices. Journal of Machine Learning Research. 2010;11(80):2287–322. Available from: http://jmlr.org/papers/v11/mazumder10a.html

15. Dahl A, Iotchkova V, Baud A, Johansson Å, Gyllensten U, Soranzo N, et al. A multiple-phenotype imputation method for genetic studies. Nature Genetics. 2016 Apr 1;48(4):466–72. Available from: https://doi.org/10.1038/ng.3513

16. Hormozdiari F, Kang EY, Bilow M, Ben-David E, Vulpe C, McLachlan S, et al. Imputing phenotypes for genome-wide association studies. The American Journal of Human Genetics [Internet]. 2016 Jul 7;99(1):89–103. Available from: https://doi.org/10.1016/j.ajhg.2016.04.013

17. LeCun Y, Bengio Y, Hinton G. Deep learning. Nature [Internet]. 2015 May 1;521(7553):436–44. Available from: https://doi.org/10.1038/nature14539

18. Kingma DP, Welling M. An introduction to variational autoencoders. Foundations and Trends® in Machine Learning. 2019;12(4):307–92. Available from: http://dx.doi.org/10.1561/2200000056

19. Qiu YL, Zheng H, Gevaert O. Genomic data imputation with variational auto-encoders. GigaScience. 2020 Aug;9(8). Available from: https://doi.org/10.1093/gigascience/giaa082

20. Nazábal A, Olmos PM, Ghahramani Z, Valera I. Handling incomplete heterogeneous data using VAEs. Pattern Recognition. 2020;107:107501. Available from: https://www.sciencedirect.com/science/article/pii/S0031320320303046

21. Goodfellow I, Pouget-Abadie J, Mirza M, Xu B, Warde-Farley D, Ozair S, et al. Generative adversarial nets. In: Ghahramani Z, Welling M, Cortes C, Lawrence N, Weinberger KQ, editors. Advances in neural information processing systems. Curran Associates, Inc.; 2014. Available from: https://proceedings.neurips.cc/paper/2014/file/5ca3e9b122f61f8f06494c97b1afccf3-Paper.pdf

22. Yoon J, Jordon J, Schaar M van der. GAIN: Missing data imputation using generative adversarial nets. In: Dy J, Krause A, editors. Proceedings of the 35th international conference on machine learning. PMLR; 2018. p. 5689–98. (Proceedings of machine learning research; vol. 80). Available from: https://proceedings.mlr.press/v80/yoon18a.html

23. García-Laencina PJ, Sancho-Gómez JL, Figueiras-Vidal AR. Pattern classification with missing data: A review. Neural Computing and Applications. 2010 Mar 1;19(2):263–82. Available from: https://doi.org/10.1007/s00521-009-0295-6

24. Srivastava A, Valkov L, Russell C, Gutmann MU, Sutton C. VEEGAN: Reducing mode collapse in GANs using implicit variational learning. In: Guyon I, Luxburg UV, Bengio S,

Wallach H, Fergus R, Vishwanathan S, et al., editors. Advances in neural information processing systems. Curran Associates, Inc.; 2017. Available from: https://proceedings.neurips.cc/paper/2017/file/44a2e0804995faf8d2e3b084a1e2db1d-Paper.pdf

25. Beaulieu-Jones BK, Moore JH. MISSING DATA IMPUTATION IN THE ELECTRONIC HEALTH RECORD USING DEEPLY LEARNED AUTOENCODERS. Pacific Symposium on Biocomputing Pacific Symposium on Biocomputing. 2017;22:207–18. Available from: https://doi.org/10.1142/9789813207813_0021

26. Kollewe K, Mauss U, Krampfl K, Petri S, Dengler R, Mohammadi B. ALSFRS-r score and its ratio: A useful predictor for ALS-progression. Journal of the Neurological Sciences. 2008 Dec 15;275(1):69–73. Available from: https://doi.org/10.1016/j.jns.2008.07.016

27. Arisdakessian C, Poirion O, Yunits B, Zhu X, Garmire LX. DeepImpute: An accurate, fast, and scalable deep neural network method to impute single-cell RNA-seq data. Genome Biology. 2019 Oct 18;20(1):211. Available from: https://doi.org/10.1186/s13059-019-1837-6

28. Vincent P, Larochelle H, Bengio Y, Manzagol PA. Extracting and composing robust features with denoising autoencoders. In: Proceedings of the 25th international conference on machine learning. New York, NY, USA: Association for Computing Machinery; 2008. p. 1096–103. (ICML '08). Available from: https://doi.org/10.1145/1390156.1390294

29. Vincent P, Larochelle H, Lajoie I, Bengio Y, Manzagol PA. Stacked denoising autoencoders: Learning useful representations in a deep network with a local denoising criterion. Journal of Machine Learning Research. 2010;11(110):3371–408. Available from: http://jmlr.org/papers/v11/vincent10a.html

30. Miotto R, Li L, Kidd BA, Dudley JT. Deep patient: An unsupervised representation to predict the future of patients from the electronic health records. Scientific Reports. 2016 May 17;6(1):26094. Available from: https://doi.org/10.1038/srep26094

31. Wu R, Zhang A, Ilyas I, Rekatsinas T. Attention-based learning for missing data imputation in HoloClean. In: Dhillon I, Papailiopoulos D, Sze V, editors. Proceedings of machine learning and systems. 2020. p. 307–25. Available from: https://proceedings.mlsys.org/paper/2020/file/202cb962ac59075b964b07152d234b70-Paper.pdf

32. Spinelli I, Scardapane S, Uncini A. Missing data imputation with adversarially-trained graph convolutional networks. Neural Networks. 2020;129:249–60. Available from: https://www.sciencedirect.com/science/article/pii/S0893608020302185

33. Kyono T, Zhang Y, Bellot A, Van der Schaar M. MIRACLE: Causally-aware imputation via learning missing data mechanisms. In: Advances in neural information processing systems. Curran Associates, Inc.; 2021. Available from: https://proceedings.neurips.cc//paper/2021/hash/c80bcf42c220b8f5c41f85344242f1b0-Abstract.html

34. Dahl A, Thompson M, An U, Krebs M, Appadurai V, Border R, et al. Phenotype integration improves power and preserves specificity in biobank-based genetic studies of MDD. bioRxiv [Internet]. 2022; Available from: https://www.biorxiv.org/content/early/2022/08/15/2022.08.15.503980
