# Supplementary material for: Deep learning-based phenotype imputation on population-scale biobank data increases genetic discoveries
Source: Nat Genet. 2023 Nov 20;55(12):2269–76. doi: 10.1038/s41588-023-01558-w (PMC10703681; doi:10.1038/s41588-023-01558-w)
Supplement: Supplementary file 2 — Reporting Summary [file 41588_2023_1558_MOESM2_ESM.pdf]

nature portfolio

Corresponding author(s): Ulzee An, Sriram Sankararaman

Last updated by author(s): Aug 14, 2023

Reporting Summary

Nature Portfolio wishes to improve the reproducibility of the work that we publish. This form provides structure for consistency and transparency in reporting. For further information on Nature Portfolio policies, see our Editorial Policies and the Editorial Policy Checklist.

Statistics

For all statistical analyses, confirm that the following items are present in the figure legend, table legend, main text, or Methods section.

n/a	Confirmed	
☐	☒	The exact sample size (*n*) for each experimental group/condition, given as a discrete number and unit of measurement
☐	☒	A statement on whether measurements were taken from distinct samples or whether the same sample was measured repeatedly
☐	☒	The statistical test(s) used AND whether they are one- or two-sided<br>*Only common tests should be described solely by name; describe more complex techniques in the Methods section.*
☐	☒	A description of all covariates tested
☐	☒	A description of any assumptions or corrections, such as tests of normality and adjustment for multiple comparisons
☐	☒	A full description of the statistical parameters including central tendency (e.g. means) or other basic estimates (e.g. regression coefficient) AND variation (e.g. standard deviation) or associated estimates of uncertainty (e.g. confidence intervals)
☐	☒	For null hypothesis testing, the test statistic (e.g. *F*, *t*, *r*) with confidence intervals, effect sizes, degrees of freedom and *P* value noted<br>*Give P values as exact values whenever suitable.*
☐	☒	For Bayesian analysis, information on the choice of priors and Markov chain Monte Carlo settings
☐	☒	For hierarchical and complex designs, identification of the appropriate level for tests and full reporting of outcomes
☐	☒	Estimates of effect sizes (e.g. Cohen's *d*, Pearson's *r*), indicating how they were calculated

Our web collection on statistics for biologists contains articles on many of the points above.

Software and code

Policy information about availability of computer code

Data collection	No software was used for data collection
Data analysis	AutoComplete: https://github.com/sriramlab/AutoComplete<br>Plink 2.0: https://www.cog-genomics.org/plink/2.0/<br>LDSC: https://github.com/bulik/ldsc<br>HI-VAE: https://github.com/probabilistic-learning/HI-VAE<br>GAIN: https://github.com/jsyoon0823/GAIN<br>kNN: https://scikit-learn.org/stable/modules/generated/sklearn.impute.KNNImputer.html<br>MissForest: https://cran.r-project.org/web/packages/missForest/index.html<br>MICE: https://github.com/AnotherSamWilson/miceforest

For manuscripts utilizing custom algorithms or software that are central to the research but not yet described in published literature, software must be made available to editors and reviewers. We strongly encourage code deposition in a community repository (e.g. GitHub). See the Nature Portfolio guidelines for submitting code & software for further information.

April 2023

Data

Policy information about availability of data

All manuscripts must include a data availability statement. This statement should provide the following information, where applicable:

- Accession codes, unique identifiers, or web links for publicly available datasets
- A description of any restrictions on data availability
- For clinical datasets or third party data, please ensure that the statement adheres to our policy

The genotype and phenotype data are available by application from the UKBB https://www.ukbiobank.ac.uk. The LD Scores from the 1000 Genomes project are available from https://alkesgroup.broadinstitute.org/LDSCORE/.

Bilirubin GWAS from "Clinical Laboratory Test-Wide Association Scan of Polygenic Scores Identifies Biomarkers of Complex Disease" [72]: http://ftp.ebi.ac.uk/pub/databases/gwas/summary_statistics/GCST90012001-GCST90013000/GCST90012749/

MDD GWAS by PGC (excluding UKBB and 23andMe) from "Genome-Wide Association Analyses Identify 44 Risk Variants and Refine the Genetic Architecture of Major Depression": https://figshare.com/articles/dataset/mdd2018/14672085

MDD GWAS of 23andMe cohort from "Minimal phenotyping yields genome-wide association signals of low specificity for major depression": https://figshare.com/s/b61e44d5142cc0690772

Lifetime Cannabis use GWAS from "Genome-Wide Association Study of Lifetime Cannabis Use Based on a Large Meta-Analytic Sample of 32 330 Subjects from the International Cannabis Consortium": https://www.ru.nl/bsi/research/group-pages/substance-use-addiction-food-saf/vm-saf/genetics/international-cannabis-consortium-icc/

Research involving human participants, their data, or biological material

Policy information about studies with human participants or human data. See also policy information about sex, gender (identity/presentation), and sexual orientation and race, ethnicity and racism.

Reporting on sex and gender	The study was not specific to one sex or gender. Biological sex as collected by the UK Biobank was used as features in the imputation experiments and as covariates in genome-wide association analysis.
Reporting on race, ethnicity, or other socially relevant groupings	The study focuses on White British individuals in the UK Biobank to control for population structure in downstream genome-wide association analysis.
Population characteristics	The study included as many individuals as possible from the UK Biobank who where White and British.
Recruitment	Nearly 500,000 individuals were recruited as part of the UK Biobank effort aged between 40 and 69 in the United Kingdom. Participants agreed to share their health information through 22 centers around the country. Written consent was provided by the participants, and follow up assessments included face-to-face interviews, body measurements, and sample collection.
Ethics oversight	The Ethics Advisory Committee (EAC) provides advice to the UK Biobank Board on ethical issues that arise during the maintenance, development, and use of the UK Biobank.

Note that full information on the approval of the study protocol must also be provided in the manuscript.

Field-specific reporting

Please select the one below that is the best fit for your research. If you are not sure, read the appropriate sections before making your selection.

☒ Life sciences ☐ Behavioural & social sciences ☐ Ecological, evolutionary & environmental sciences

For a reference copy of the document with all sections, see nature.com/documents/nr-reporting-summary-flat.pdf

Life sciences study design

All studies must disclose on these points even when the disclosure is negative.

Sample size	The number of individuals in the Cardiometabolic dataset was 285,405. The number of individuals in the Psychiatric Disorders dataset was 337,126. As many individuals from the UK Biobank who were White British and unrelated were included in the study.
Data exclusions	Individuals who were closely related genetically were pruned from each dataset before all experiments. This was to maintain the validity of the genome-wide association analysis downstream.
Replication	The study finds that replication of genome-wide associations were successful for all phenotypes tested.

Randomization	Prior to the imputation simulation experiments, all individuals in each dataset was shuffled randomly such that individuals would be allocated randomly in downstream experiments.
Blinding	Due to the individuals in each dataset being shuffled, all imputation experiments were blind to which individuals would be allocated to the training, validation, or test set.

Reporting for specific materials, systems and methods

We require information from authors about some types of materials, experimental systems and methods used in many studies. Here, indicate whether each material, system or method listed is relevant to your study. If you are not sure if a list item applies to your research, read the appropriate section before selecting a response.

Materials & experimental systems

n/a	Involved in the study
☒	☐ Antibodies
☒	☐ Eukaryotic cell lines
☒	☐ Palaeontology and archaeology
☒	☐ Animals and other organisms
☒	☐ Clinical data
☒	☐ Dual use research of concern
☒	☐ Plants

Methods

n/a	Involved in the study
☒	☐ ChIP-seq
☒	☐ Flow cytometry
☒	☐ MRI-based neuroimaging
